# Impact of edentulism on community-dwelling adults in low-income, middle-income and high-income countries: a systematic review

**DOI:** 10.1136/bmjopen-2024-085479

**Published:** 2024-12-04

**Authors:** Emma Hunter, Luciana De Moura Brito, Prabhath Piyasena, Egle Petrauskiene, Nathan Congdon, Georgios Tsakos, Gianni Virgili, Manu Mathur, Jayne V Woodside, Cláudio Leles, Gerald McKenna

**Affiliations:** 1 Centre for Public Health, Queen's University Belfast, Belfast, UK; 2 Federal University of Goiás, Goiânia, Brazil; 3 Vision and Eye Research Institute, Anglia Ruskin University Faculty of Health and Social Care, Chelmsford, UK; 4 Department of Epidemiology and Public Health, UCL, London, UK; 5 Ophthalmology and Public Health, Queen's University Belfast, Belfast, Antrim, UK; 6 Orbis International, New York, New York, USA; 7 NEUROFARBA, University of Florence, Firenze, Toscana, Italy; 8 IRCCS, Fondazione Bietti, Roma, Italy; 9 Queen Mary University of London, London, UK; 10 Department of Reconstructive Dentistry and Gerodontology, School of Dental Medicine, University of Bern, Bern, Switzerland; 11 University of Zurich Center of Dental Medicine, Zurich, Switzerland

**Keywords:** Public Health, Health, Systematic Review

## Abstract

**Abstract:**

**Objectives:**

This study aims to comprehensively explore the consequences of edentulism on community-dwelling adults in low-income, middle-income and high-income countries.

**Design:**

Systematic review and Synthesis Without Meta-Analysis (SWiM).

**Data sources:**

MEDLINE, Embase, Scopus, Web of Science and the Cochrane Central Register of Controlled Trials (CENTRAL) were searched from inception to 21 March 2023, in addition to grey literature searches, reference cross-checking and study recommendations.

**Eligibility criteria:**

Interventional and observational epidemiological studies of community-dwelling completely edentulous adults aged 18 years and above, residing in low-income, middle-income and high-income countries were included. Animal studies, studies of children and adolescents, studies of exclusively non-community-dwelling individuals and studies exclusive to partially dentate, dentate and treated edentulous individuals were excluded. There were no language restrictions. Search terms were designed to identify relevant articles, which examined the impact of edentulism on oral health-related quality of life, frailty, general health, physical health, mental health, nutritional status, employability, education, socioeconomic status and mortality.

**Data extraction and synthesis:**

Data were extracted manually by two authors. Risk of bias was assessed using the QualSyst Tool. Articles were synthesised and reported using SWiM guidelines.

**Results:**

The seven broad outcome areas included mortality, frailty, mental health, general health, quality of life, health behaviour and nutrition. We identified no studies assessing the impact of edentulism on productivity or other economic outcomes, and no randomised trials of treatment for edentulism with any of these outcomes. Among 364 articles identified from database searches and 38 additional articles from reference cross-checks and professional recommendations, title and abstract screening resulted in a full text review of 58. Among these, 32 were subsequently included for narrative synthesis, with sample sizes ranging from 539 to 237 023 participants. All studies (n=32) contributing to the synthesis reported negative impact of edentulism on outcomes including mortality, frailty, mental health, general health, cognition and nutrition.

**Conclusions:**

Edentulism has a consistently negative impact on the health outcomes examined in this review. Randomised trials are needed to evaluate interventions reducing the burden of edentulism, specifically with regard to economic and productivity outcomes.

**PROSPERO registration number:**

CRD42022320049.

**STRENGTHS AND LIMITATIONS OF THIS STUDY**

- This systematic review was strengthened by a comprehensive search strategy, which placed no restrictions on language or publication date.
- The quality of each study was assessed using the QualSyst Tool Standardised Checklist for Quantitative Studies for Quantitative Research.
- The majority of studies included were cross-sectional and no relevant interventional studies were identified for inclusion within the review, limiting the ability to establish causality.
- As no studies exploring the socioeconomic impacts of edentulism were identified within the search strategy, future systematic reviews in this area should consider searching databases relevant to economics and social sciences.
- Due to heterogeneity across the included studies, meta-analysis could not be performed and narrative synthesis was completed using Synthesis Without Meta-Analysis reporting guidelines.

## Introduction

 Edentulism, an adverse oral health outcome characterised by the loss of all natural teeth,^1^ is among the most common global causes of disability.^2^ It is an end-stage, irreversible but highly treatable and largely preventable oral health status, which is primarily caused by untreated dental caries and periodontal disease, in addition to dental trauma in some cases.^3^

The global prevalence of edentulism is generally declining^2^; however, according to the WHO Oral Health Country/Area Profile Project, there are wide disparities in prevalence between high-income countries (HICs) and low-income and middle-income countries (LMICs). Available figures indicate that edentulism is decreasing in HICs but increasing in LMICs, primarily due to population growth and ageing in the latter.^4^ In 2009, 15% of persons aged 65 years in the UK were edentulous,^5^ compared with 63.2% of those aged 62 years in Brazil in 2005.^6^ A recent systematic review and meta-analysis has indicated the global prevalence of edentulism in community-dwelling adults aged 45 years or older ranges between 1.1% and 70%. Edentulism prevalence was highest (32%) in countries with the lowest gross national income, but with significant intracountry variation, indicating that edentulism is a persistent and complex issue with a significant health burden on the global population.^7^

Edentulism is also a disease characterised by social inequalities, linked to limited educational attainment and low income.^8 9^ The Centre for Disease Control and Prevention 2011–2016 Oral Health Surveillance report from the USA showed a 34% prevalence of edentulism among those considered poor, with a household income level below 100% of the federal poverty level, compared with 11% of those not so classified.^10^ The same report found a 35% prevalence among those with less than a high school education compared with only 9% among those educated beyond high school. Edentulism is a condition that disproportionately affects older adults, highlighted in a 2018 study showing that prevalence of edentate Indonesian adults aged over 80 years of age was 29.8%, compared with 7.2% of the entire adult population.^9^

Existing systematic reviews have reported an increased risk of mortality among edentulous persons.^11^ This may be mediated by the negative impact of edentulism on nutrition, though evidence for this has been inconclusive in some reviews.^12^ No systematic review examining the impact of the untreated edentulous adult patient, without age group restriction, on a wide range of health and social impacts was identified prior to the commencement of this review. The WHO Global Strategy on Oral Health is aimed to encourage oral health promotion, improved access to care and reduction of the burden and impact of oral diseases.^13^ Mobilisation of resources to achieve these goals at the national and regional level depends heavily on high-quality evidence on the distribution, social determinants and impacts of edentulism on health and livelihood. As clearly outlined in the WHO Oral Health Action Plan 2023–2030,^13^ this should also be fully integrated within health systems. Including evidence for those populations where the need is greatest is crucially important to drive investment of resources where required, and many existing reviews have not included, or have not focused on, data from LMICs.

The aim of the current systematic review is to summarise available evidence on the global impacts of edentulism, in order to support future randomised trials assessing the health and economic benefits of low-cost management strategies. This work is modelled on studies in the eyecare sector,^14^ where trials demonstrating the workplace productivity benefits of low-cost interventions such as the provision of glasses have helped to drive investment from governments and industry in these services.

## Methods

### Registration and protocol

Registration with PROSPERO^15^ for this systemic review was completed on 31 May 2022 (registration number CRD42022320049). A protocol for the systematic review was designed, using the Preferred Reporting Items for Systematic Reviews and Meta-Analyses (PRISMA) 2020 checklist.^16^

### Search strategy and sources of evidence

A comprehensive search strategy was designed in collaboration with the support of a subject matter librarian and experienced researchers in the fields of dental public health, gerodontology, nutrition and medicine, in addition to implementing elements of the Cochrane Effective Practice and Organisation of Care LMIC Filters 2020.^17^ A population, exposure, outcome (PEO)-focused question was formed:

- Population: community-dwelling adults aged 18 years and over, residing in low-income, middle-income and high-income countries.
- Exposure: edentulism or loss of all natural teeth.
- Outcome: impact of edentulism on income, socioeconomic status, work productivity, education, health outcomes, nutrition, mortality, frailty and oral health-related quality of life.

Five databases were searched for this systematic review: MEDLINE, EMBASE, Scopus, Web of Science and the Cochrane Central Register of Controlled Trials (CENTRAL). Separate search strategies were used for MEDLINE, EMBASE, Web of Science, Scopus and the Cochrane Register of Controlled Trails (CENTRAL). A combination of medical subject headings and keywords classified under the general (all fields) category were searched. The search terms used, restrictions applied and final search dates for the database searches are displayed in online supplemental appendix 1. Grey literature was searched. Members of the systematic review team with professional expertise in the area of edentulism (EP, GM) were contacted for additional suggestions of suitable literature for inclusion.

For any data missing from included studies, efforts were made to contact the authors.

### Inclusion and exclusion criteria

Interventional and observational epidemiological studies of community-dwelling, completely edentulous adults aged 18 and over residing in low-income, middle-income and high-income countries were included. Studies exclusive to partially or fully dentate people and previously treated edentulous patients were excluded, as were studies of children and adolescents, non-community-dwelling individuals, animals and non-primary research (online supplemental appendix 2).

### Study selection

There was an initial assessment and removal of duplicate articles. Title and abstract screening of identified literature was completed by a single reviewer (EH) using the defined inclusion and exclusion criteria, and PRISMA 2020 guidelines^15^ were applied to the study selection. This was followed by screening of full-text articles by two masked and independent reviewers (EH and LDMB). In case of disagreements between the reviewers, consensus was sought through discussion. For persistent disagreements, a senior member of the review team (GMK) made the final decision regarding study inclusion or exclusion. Articles were searched for and located electronically. All studies meeting the inclusion criteria were selected for full text review, in addition to any studies identified following reference checks or professional recommendations.

### Review outcomes

The anticipated primary outcomes of interest for this systematic review were the impact of edentulism on the following factors: oral health-related quality of life, frailty, economic productivity, general health, physical health, mental health, nutritional status and mortality.

### Data extraction

Full-text articles were obtained in order to complete data extraction. Data (online supplemental appendix 3) were collected by two independent, masked investigators (EH and LD). In the case of disagreements about data collection, consensus was sought between the reviewers before data extraction was finalised. In the event of persistent disagreements, a member of the systematic review team (EP) was available to make the final decision on data extraction. In the event of missing information or lack of clarity, study authors were contacted by email, and the article excluded from data extraction in the event of non-response.

### Risk-of-bias assessment

The risk of bias of all studies included for analysis following data extraction was evaluated using the QualSyst Tool Standardised Checklist for Quantitative Studies for Quantitative Research.^18^ checklist items (online supplemental appendix 4) were used to assess particular elements of each study, with a score of yes (2), partial (1) and no (0) awarded for each question. A score was generated, which was calculated by dividing the total score awarded by the maximum score possible for the particular study design. Studies with a score<50% were interpreted as ‘poor’, 50%–70% as ‘adequate’, 71%–79% as ‘good’ and ≥80% were considered ‘strong’.^19^ The maximum possible score was 28. Three criteria (criteria 5, 6 and 7) regarding allocation of participants, masking of investigators and masking of participants, were applicable to interventional studies only and were excluded for observational studies, for which the maximum score was 22. Two masked reviewers (EH and LDMB) independently assessed the quality of each study qualifying for analysis using one standardised checklist for all primary quantitative research papers, and a score was calculated. For any disputed scores, a discussion was held between the reviewers to achieve a consensus score, with another member of the research team mediating to resolve conflicts; final scores were validated by this member (PP).

### Results synthesis

A Kappa Statistic was calculated using SPSS Statistics, V.27, to assess the degree of interinvestigator agreement for articles selected for final inclusion following full title screening.

### Synthesis Without Meta-Analysis (SWiM)

Due to high heterogeneity between the included studies, it was not possible to perform a meta-analysis within this review. As an alternative, SWiM has been performed.^20^ The exposure of edentulism was categorised as harmful or beneficial to the outcomes identified. As per Cochrane Guidance,^20 21^ the direction of the effect has been reported using the adjusted estimates for each study, without respect to measures of statistical significance. Any studies with conflicting results were assessed as per SWiM guidance to determine whether>70% of outcomes reported a consistent directionality. If ≤70% of results reported a consistent direction of effect, the studies would be classed as inconsistent and excluded from the synthesis. Risk of bias is presented in the SWiM table,^21^ with studies graded as strong (≥80%) having ‘low risk of bias’, studies graded as good (71%–79%) and adequate (50%–70%) having ‘some concerns’ and any studies with a QualSyst score<50% considered at ‘high risk of bias’.

### Patient and public involvement

None.

## Results

**Figure 1:**
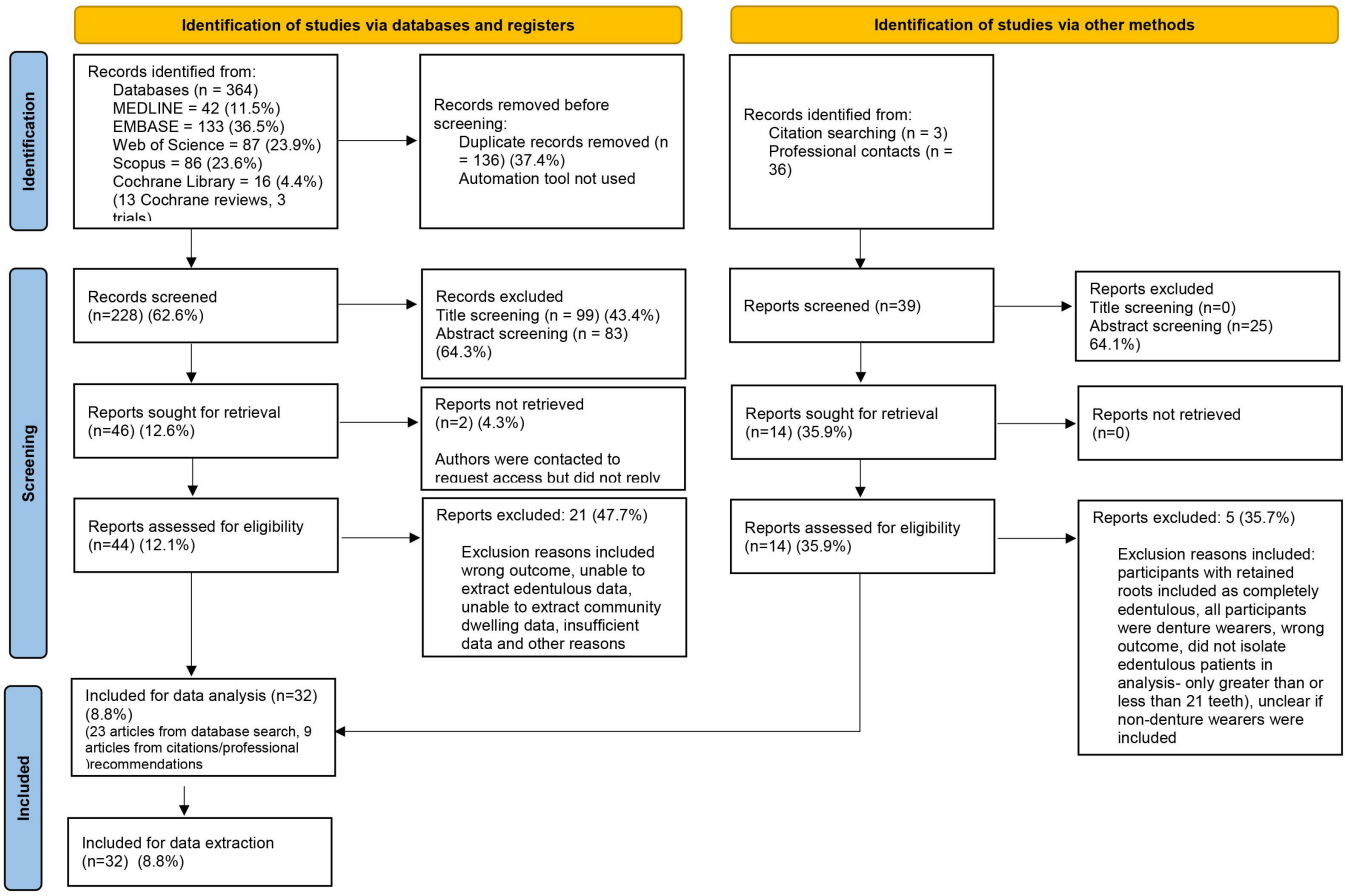
Preferred Reporting Items for Systematic Reviews and Meta-Analyses flowchart.^15^

Among 364 articles identified through database searches, following removal of duplicates, 261 (71.7%) were included for title and abstract review by one researcher (EH). Following removal of articles not relevant to the systematic review questions, 44 articles (12.1%) from the original search underwent full text screening. Reference cross-checks and hand searches were then performed, in addition to consultations with professional contacts, resulting in an additional 36 articles being identified, among which 11 (30.6%) underwent full text review. The final total of articles undergoing full text analysis was 55, resulting in 32 articles (58.2%) being included for data extraction (figure 1). Agreement between the researchers in the selection of studies for the full text analysis was substantial (kappa=0.7660).^22^ Reasons for disagreement included varying definitions of ‘community-dwelling participants’ and differing opinions on whether the outcome of an article was appropriate for study inclusion.

### Characteristics of excluded studies

During title screening of articles obtained from databases, 99 were excluded (online supplemental appendix 5). Following abstract review, a further 108 studies, from the database, citation searches and professional recommendations were excluded (online supplemental appendix 6). A total of 60 studies were eligible for full text review; however, 2 article full texts could not be accessed, resulting in full text review of 58 studies, among which, 26 studies were subsequently excluded (online supplemental appendix 7), leaving 32 studies available for data extraction (online supplemental appendix 8).

### Studies included in narrative review

The characteristics of the 32 studies^223^,^53^ included in the narrative review are summaried in online supplemental file 8. 9 of the included studies employed cohort designs,^23 28 29 32 35 36 43 44 49^ 1 a case-cohort design^27^ and 22 studies were cross-sectional.^224^,^26 30 31 33 34 37^ There were no interventional studies included in the final review. The range of year of publication of primary studies was 2000 to 2022. The patient age groups for included studies ranged from a minimum of 18 years to a maximum of >85 years.

A broad range of outcomes were identified as follows: frailty (n=13),^23^,^3453^ mental health (n=5),^239^,^42^ general health problems (n=5),^35^,^3844^ mortality (n=4)^29 43 45 46^ quality of life (n=3),^2 47 48^ disordered sleep (n=2),^50 52^ sedentary behaviour (n=1)^51^ and nutrition (n=1).^49^ No studies were identified with outcomes of productivity, income or other economic metrics.

Studies in the current review included patients recruited from LMICs (n=14)^24^,^2731 34 39 40 42 47 48 51^ and HICs (n=15),^2328^,^30 32 33 35^ as determined by the World Bank Atlas.^54^ One study focused on both low-income and high-income countries^43^ and two studies recruited eligible patients across all country income levels.^2 41^ The sample sizes of included studies ranged from 539 to 237 023 participants. The percentage of edentulous participants in each study ranged from 2.8% to 44.8%, while one study^46^ had only edentulous participants. This is consistent with the published literature, which has indicated a global edentulism prevalence of 1.1% to 70%.^7^

### Risk-of-bias assessment

The QualSyst tool design, non- interventional studies have three assessment domains excluded from their risk of bias total scores.^18^ In accordance with the QualSyst scoring system used, 81.3% (n=27) of studies were graded as strong (≥80%) and 12.5% (n=4) as good (71%–79%) while one study was graded as adequate (50%–70%) (online supplemental appendix 9).

### Narrative synthesis

Due to heterogeneity of outcomes and inconsistencies in the measures of effect identified within this review, meta-analysis was not possible. Narrative synthesis was performed according to the SWiM reporting guidelines.^20^ Studies were grouped into seven outcome groups for comparison and synthesis: frailty, general health, mental health, mortality, quality of life, nutrition and health behaviour (online supplemental appendix 10). No relevant studies were identified for inclusion in the synthesis with outcomes of productivity, income or other economic impacts. Direction of effect in conjunction with study size and quality is displayed in a direction of effect plot, following guidance from Boon and Thompson^21^ (table 1). All studies contributed to the narrative synthesis and all studies indicated that edentulism had a negative impact on the outcomes identified. Three studies^29 33 34^ were conducted with edentulous participants as the reference category, and the direction of effect of the impact of edentulism has been reported as reported by primary study authors without adjusting. Takata *et al*^33^ demonstrated some conflicting results, however≥70% of observations for edentulous patients had a consistent direction of effect. Most studies had a low risk of bias (n=27, 84.4%), while five studies (15.6%) warranted some concerns regarding risk of bias.

**Table 1:** Synthesis without meta-analysis effect direction

Outcome	Study	Study design	Direction of effect	Risk of bias
Health behaviour	Vancampfort, 2017^51^	Cross	▼	Low
	Smith *et al*, 2022^52^	Cross	▼	Low
	Koyama *et al*, 2018^50^	Cross	▼	Low
Quality of life	Hewlett *et al*, 2015^47^	Cross	▼	Low
	Medina-Solis *et al*, 2019^48^	Cross	▼	Low
	Tyrovolas *et al*, 2016^2^	Cross	▼	Low
Mortality	Palmer *et al*, 2015^43^	Coh	▼	Low
	Matsuyama *et al*, 2017^29^	Cross	▼	Low
	Sabbah *et al*, 2020^46^	Cross	▼	Low
	Yu *et al*, 2021^45^	Cross	▼	Low
Mental health	Koyanagi *et al*, 2018^40^	Cross	▼	Low
	Koyanagi *et al*, 2021^39^	Cross	▼	Low
	Tyrovolas *et al*, 2016^2^	Cross	▼	Low
	Vancampfort *et al*, 2017^41^	Cross	▼	Low
	Vancampfort *et al*, 2017^42^	Cross	▼	Low
General health	Barros *et al*, 2013^35^	Coh	▼	Low
	Philips *et al*, 2021^36^	Coh	▼	Low
	Heitmann *et al*, 2008^44^	Coh	▼	Low
	Lee *et al*, 2006^38^	Cross	▼	Some concerns
	Sanders *et al*, 2016^37^	Cross	▼	Some concerns
Frailty	Vélazquez-Olmedo *et al*, 2021^27^	CaCo	▼	Low
	Matsuyama *et al*, 2017^29^	Coh	▼	Low
	Ramsay *et al*, 2017^28^	Coh	▼	Low
	Ritchie *et al*, 2000^32^	Coh	▼	Some concerns
	Vancampfort *et al*, 2019^25^	Cross	▼	Low
	Vancampfort *et al*, 2017^26^	Cross	▼	Low
	Arokiasamy *et al*, 2018^24^	Cross	▼	Low
	Huang *et al*, 2022^53^	Cross	▼	Low
	de Andrade *et al*, 2013^34^	Cross	▼	Low
	Avlund *et al*, 2011^23^	Cross	▼	Some concerns
	Gu *et al*, 2019^31^	Cross	▼	Low
	Albani *et al*, 2021^30^	Cross	▼	Low
	Takata *et al*, 2004^33^	Cross	▼	Low
Nutrition	Kiesswetter *et al*, 2018^49^	Coh	▼	Some concerns

Table adapted from Boon and Thomson.^21^

Risk of bias (RoB): Sstrong≥80% scores=low RoB, good 71%–79%, adequate 50%–70%=some concerns, ≤50%=high concerns.

Effect direction: upward arrow (▲)=positive health impact, downward arrow (▼)=negative health impact, sideways arrow (◄►)=no change/mixed effects/conflicting findings.

Sample size: Ffinal sample size (individuals) in intervention group, Llarge arrow (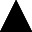
▲)>300; medium arrow (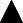
▲) 50–300; small arrow (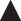
▲)<50.

CaCocase–cohortCohcohortCrosscross-sectional

Data extracted from included studies is available in online supplemental appendix 10 and the SWiM analysis is available in table 1.

### Frailty

13 studies^23^,^3453^ examined the relationship between edentulism and outcomes related to frailty, including frailty, handgrip strength, physical activity, weight loss, fatigue, physical fitness and functional disability. Six studies^2328^,^30 32 33^ were based in HICs and seven studies were conducted in LMICs.^24^,^2731 34 53^ One study ^33^ reported some inconsistent results but as consistency was noted in over 70% of outcomes, it was included within the analysis and the consensus was reached on the direction of effect. Edentulism had a negative impact on frailty in 100% of studies included for synthesis (n=13).

### General health

Five studies,^35^,^3844^ all conducted in HICs, examined the impact of edentulism on general health outcomes, including chronic obstructive pulmonary disease (COPD), undiagnosed diabetes mellitus, obstructive sleep apnoea (OSA) and fatal and non-fatal cardiovascular disease (CVD), coronary heart disease (CHD) and stroke. All studies compared edentulous to non-edentulous patients. Edentulism had a negative impact on general health in 100% of these studies.

### Mental health

Five studies^239^,^42^ examined the impact of edentulism on mental ill-health, including mild cognitive impairment, subjective cognitive complaints, perceived stress, anxiety and depression. Three studies^39 40 42^ were based in LMICs, while the remaining two studies^2 41^ were conducted across low-income, middle-income and high-income countries. All of these studies reported a negative association between edentulism and mental health.

### Mortality

Four studies^29 43 45 46^ assessed the impact of edentulism on mortality. Three studies^29 45 46^ examined the relationship between edentulism and all-cause mortality, while one study^43^ focused on all-cause and cardiovascular mortality in people with end stage kidney disease treated with haemodialysis. Three studies were conducted in HICs,^29 45 46^ while one study^43^ was based in both low-income and high-income countries. Edentulism had a negative impact on mortality in 100% of studies.

### Quality of life

Three studies^2 47 48^ had primary outcomes related to quality of life, including subjective well-being, self-reported health status and self-rated health. Two studies were based in middle-income countries^47 48^ and one study was conducted in low-income, middle-income and high-income countries.^2^ Edentulism had a negative impact on quality of life in 100% of these studies.

### Nutrition

One study,^49^ conducted in a HIC, examined the relationship between edentulism and nutrition. In this study, edentulism had a negative impact on nutrition.

### Health behaviours

Three studies^50^,^52^ investigated the relationship between edentulism and health behaviours, including disordered sleep, sleep problems and sedentary behaviour. One study^50^ was based in an HIC, while two studies^51 52^ were based in LMICs. Edentulism had a negative impact on health behaviour in 100% of these studies.

## Discussion

This systematic review investigates the broad impacts of edentulism on frailty, general health, mental health, mortality, quality of life, nutrition and health behaviour in the context of low-income, middle-income and high-income countries. All (100%) studies included in the synthesis reported that edentulism had a negative association with measured outcomes. Studies carried out in HICs largely investigated frailty (n=6, 40%), mortality (n=3, 20%) and non-communicable disease outcomes (n=5, 33.3%), including undiagnosed diabetes mellitus, CVD, CHD, stroke history, COPD and OSA. LMIC based studies focused particularly on frailty outcomes (n=7, 50%) and mental health outcomes (n=3, 21.4%), including cognitive impairment, stress and depression.

The studies presented reveal that edentulism has a considerable impact on the examined health outcomes. Matsuyama *et al*^29^ observed that dentate people had a longer life expectancy, healthy life expectancy and shorter life expectancy with disability for both men and women compared with edentulous participants. Differences were also noted between denture wearers and non-denture wearers in a longitudinal analysis by Sabbah *et al*^46^ which described a differential burden of mortality risk among edentulous denture wearers versus non-denture wearers in an American study of 1649 adults. Edentulous denture wearers were found to have reduced risk of all-cause mortality (HR 0.85 95% CI 0.73 to 0.98) compared with edentulous non-denture wearers, though cardiovascular death (HR 0.84 95% CI 0.67 to 1.06) and cancer mortality (HR 0.87 95% CI 0.64 to 1.17) did not differ between the groups. A study across low-income, middle-income and high-income countries conducted by Tyrovolas *et al*^2^ reported a positive correlation between edentulism and depression, which was statistically significant in the <50 age group (OR 1.57 95% CI 1.23 to 2.0), suggesting that younger edentulous people may be more likely to experience depression, possibly due to impacts on self-esteem and/or stigma related to severe tooth loss at an earlier age.

To our knowledge, this is the first review that is focused on the wide-ranging impacts of edentulism on both health and sociodemographic outcomes. The strengths of the current review lie in the rigorous methodology used, in accordance with PRISMA guidelines.^15^ This is supported by a focused ‘PEO’ question. The search strategy, in accordance with Cochrane guidance,^55^ placed no language or date restrictions on the literature search, ensuring the review minimised bias and reduced the probability of omitting relevant research.^56^ Despite inclusion of search terms to identify studies with sociodemographic outcomes of employability, socioeconomic status and education, no studies were identified within this review. If this review were to be repeated, consideration towards searching economic and social science databases may help to identify studies with economic outcomes.

This systematic review is strengthened by relatively large sample sizes in included studies, which should improve the representativeness of the data, enhance the accuracy of observed mean values and provide narrower confidence limits.^57^ The samples have similar male/female ratios except for one study which included male participants only.^28^

Over half (53%)^224^,^26 29 31 39^ of included studies relied on self-reported data for the exposure variable (edentulism). The benefits of self-reported data include that it generally allows efficient and cost-effective collection of large quantities of data.^58^ The literature also supports the validity of self-reported dental status.^59^ Douglass *et al* have reported no significant differences between teeth counting by oral examination versus self-report.^60^ However, the potential for inaccuracy or bias cannot be discounted.

This review was limited by a lack of high-quality interventional studies suitable for inclusion. A high proportion of studies included in the review are cross-sectional in design (n=22), which therefore limits our review both in terms of establishing causality and in the ability to observe the relationship between edentulism and its health and social impacts over time.

Meta-analysis was deemed to be unsuitable for this review due to heterogeneity between studies in terms of design, effect measures and study outcomes. Synthesis was performed using SWiM.^20^ Limitations of this method include a failure to account for differences in sample size between studies, and a lack of information on the magnitude of effect sizes.^20^ This approach also sacrifices statistical power when compared with meta-analytic approaches.^61^

However, after being unable to obtain the full texts for two studies through the usual routes and after attempts to contact the authors, two studies were excluded from the review.

The intention at the protocol stage was to exclude any edentulous participants who had undergone treatment. Due to the data collection protocols in a number of studies, the treatment status of edentulous participants was unclear in all but one report.^46^ One study was also unclear across all outcomes as to whether participants were entirely or partially (0–4 teeth) edentulous,^44^ and one study failed to specify if any edentulous participants had retained roots.^32^ The result of these shortcomings is our inability to exclude the possibility that some treated edentulous participants might have been included in the review.

For each broad outcome area, there is an uneven distribution of country income levels across included papers, which may limit the generalisability of the study. Studies exploring general health outcomes were sourced exclusively from HICs, while mental health outcomes were obtained solely from studies based in LMICs.

While our review demonstrated reasonably even representation from LMICs (n=14) and HICs (n=15), due to the disparity of edentulism prevalence skewed towards LMICs, inclusion of additional papers from LMICs would strengthen the relevance to the countries most impacted by edentulism.

A lack of research in this area has been highlighted by our finding limited evidence covering a variety of health outcomes related to edentulism, and a complete lack of data for socioeconomic outcomes, including work productivity and income. There was also a lack of studies providing clearly defined comparisons between treated and untreated edentulous patients.

Future investigation in this field would benefit from increased representation from LMICs, more work on the economic impacts of edentulism and additional research on age stratified impact of edentulism (50–65, 65–75 and 75 years and above) to reflect the ageing of populations in both LMICs and HICs. The importance of further research in this area is underscored by the growth and ageing of the global population, with projections by the United Nations indicating that it will reach 8.6 billion by 2030,^62^ as the number of people over the age of 60 years doubles by 2050.

The evidence presented in this review suggests a link between edentulism and a range of undesirable health outcomes. Policymakers should consider the importance of prevention of edentulism with an aim to maintain good oral health throughout the life course to prevent excessive tooth loss. The WHO has recognised the significant burden of poor dental health and as a result has recently developed the WHO Global Strategy on Oral Health.^13^ To achieve these ambitious goals will require strengthening the dental workforce and aggressive target setting as part of achieving global universal health coverage. A crucial step in mobilising the policy support for these investments in oral health at the national and global level will be high-quality evidence of the economic impacts of edentulism, particularly in LMICs, the setting in which this impairment remains most prevalent.

## supplementary material

10.1136/bmjopen-2024-085479 online supplemental file 1

10.1136/bmjopen-2024-085479 online supplemental file 2

10.1136/bmjopen-2024-085479 online supplemental file 3

10.1136/bmjopen-2024-085479 online supplemental file 4

10.1136/bmjopen-2024-085479 online supplemental file 5

10.1136/bmjopen-2024-085479 online supplemental file 6

10.1136/bmjopen-2024-085479 online supplemental file 7

10.1136/bmjopen-2024-085479 online supplemental file 8

10.1136/bmjopen-2024-085479 online supplemental file 9

10.1136/bmjopen-2024-085479 online supplemental file 10

## Data Availability

Data are available on reasonable request.
