# Supplementary material for: Impact of edentulism on community-dwelling adults in low-income, middle-income and high-income countries: a systematic review
Source: BMJ Open. 2024 Dec 4;14(12):e085479. doi: 10.1136/bmjopen-2024-085479 (PMC11624734; doi:10.1136/bmjopen-2024-085479)
Supplement: online supplemental file 1 [file bmjopen-14-12-s001.pdf]

Appendix 1: Database Search Strategies

1. **Medline Search Strategy**

PEO Term	Database	MeSH or Keyword	Search Terms Used
Population	MEDLINE	MeSH Terms	Developed countries
	MEDLINE	Keyword Terms	high income countr*; rich countr*; wealthy countr*; developed countr*; high income nation; rich nation; developed nation; high income region; rich region; wealthy region; developed region; middle income countr*; middle income region; middle income nation; low and middle income countr*; low and middle income region; low and middle income nation; LMIC; LAMIC; low income countr*; low income nation; low income region; developing countr*; developing nation; developing region; third world countr*; third world region; third world nation; poor countr*; poor nation; poor region; deprived countr*; deprived nation; deprived region; underserved countr*, underserved nation; underserved region transitional countr*; transitional nation; transitional region
Exposure Terms	MEDLINE	MeSH Terms	Jaw, edentulous; mouth, edentulous
		Keyword Terms	Edentate; total tooth loss, edentul

Outcome Terms	MEDLINE	MeSH Terms	Socioeconomic factors; social class; income; salaries and fringe benefits; educational status; employment; nutritional status; eating; anthropometry; body fat distribution; body mass index; body size; body surface area; waist-hip ratio; frailty; sedentary behaviour; mortality; premature mortality; death; health status; health status disparities
		Keyword Terms	Socioeconomic status; socioeconomic position; wealth; subjective social status; educational level; educational attainment; employability; disability; dietary intake; productivity; handgrip strength; handgrip weakness; slowness; exhaustion; low energy; low physical activity; unintentional weight loss; health inequalities; excess mortality; premature death; excess death; oral health related quality of life.
Search Restrictions	MEDLINE		Human studies only;<br>Age restrictions:<br>Adults 19-44,<br>Young adult and adult 19-24, 19-44,<br>Middle age 45-64,<br>Middle age 45 plus years,<br>All aged 65 and over,<br>Aged 80 and over;<br>No language restrictions;<br>No date restrictions;
Final Search Date	MEDLINE		21.03.23

2. Embase Search Strategy

#	Query
1	edentulousness/
2	edentul*.mp.
3	edentate.mp.
4	total tooth loss.mp.
5	social class/
6	socioeconomic factors.mp.
7	socioeconomic position.mp. or social status/
8	subjective social status.mp.
9	socioeconomic status.mp.
10	"salary and fringe benefit"/ or salary/
11	income/ or income inequality/
12	wealth.mp.
13	educational status/ or educational attainment.mp.
14	education level.mp.
15	employment/
16	employability/
17	disability/
18	frailty/
19	slowness/
20	exhaustion/
21	low energy.mp.
22	sedentary behaviour.mp.
23	productivity/
24	handgrip strength.mp.
25	handgrip weakness.mp.
26	low physical activity.mp.

27	eating/ or dietary intake/
28	nutritional status/
29	anthropometry/
30	body fat distribution/
31	body mass index.mp.
32	body size/
33	body surface area.mp.
34	waist hip ratio/
35	unintentional weight loss.mp.
36	health status disparities.mp.
37	health disparity/ or health inequalities.mp.
38	health status/
39	oral health related quality of life.mp.
40	excess mortality/ or mortality/ or premature mortality/
41	death/
42	premature death.mp.
43	excess death.mp.
44	high income country/ or high income countr*.mp.
45	high income region.mp.
46	high income nation.mp.
47	rich countr*.mp.
48	rich region.mp.
49	rich nation.mp.
50	wealthy countr*.mp.
51	wealthy nation.mp.
52	wealthy region.mp.
53	developed country/ or developed countr*.mp.
54	developed nation.mp.
55	developed region.mp.
56	middle income country/ or middle income countr*.mp.
57	middle income region.mp.

58	middle income nation.mp.
59	(low and middle income countr*).mp. [mp=title, abstract, heading word, drug trade name, original title, device manufacturer, drug manufacturer, device trade name, keyword heading word, floating subheading word, candidate term word]
60	(low and middle income region).mp. [mp=title, abstract, heading word, drug trade name, original title, device manufacturer, drug manufacturer, device trade name, keyword heading word, floating subheading word, candidate term word]
61	(low and middle income nation).mp. [mp=title, abstract, heading word, drug trade name, original title, device manufacturer, drug manufacturer, device trade name, keyword heading word, floating subheading word, candidate term word]
62	LAMIC.mp.
63	LMIC.mp.
64	low income country/ or low income countr*.mp.
65	low income region.mp.
66	low income nation.mp.
67	developing countr*.mp.
68	developing nation.mp.
69	developing region.mp.
70	third world countr*.mp. [mp=title, abstract, heading word, drug trade name, original title, device manufacturer, drug manufacturer, device trade name, keyword heading word, floating subheading word, candidate term word]
71	third world nation.mp.
72	third world region.mp.
73	poor countr*.mp. [mp=title, abstract, heading word, drug trade name, original title, device manufacturer, drug manufacturer, device trade name, keyword heading word, floating subheading word, candidate term word]
74	poor nation.mp.
75	poor region.mp.

76	deprived countr*.mp. [mp=title, abstract, heading word, drug trade name, original title, device manufacturer, drug manufacturer, device trade name, keyword heading word, floating subheading word, candidate term word]
77	deprived nation.mp.
78	deprived region.mp.
79	underserved countr*.mp. [mp=title, abstract, heading word, drug trade name, original title, device manufacturer, drug manufacturer, device trade name, keyword heading word, floating subheading word, candidate term word]
80	underserved nation.mp.
81	underserved region.mp.
82	transitional countr*.mp.
83	transitional nation.mp.
84	transitional region.mp.
85	global.mp. or global health/ or global disease burden/
86	globalisation.mp.
87	developing.mp.
88	developed.mp.
89	1 or 2 or 3 or 4
90	5 or 6 or 7 or 8 or 9 or 10 or 11 or 12 or 13 or 14 or 15 or 16 or 17 or 18 or 19 or 20 or 21 or 22 or 23 or 24 or 25 or 26 or 27 or 28 or 29 or 30 or 31 or 32 or 33 or 34 or 35 or 36 or 37 or 38 or 39 or 40 or 41 or 42 or 43
91	44 or 45 or 46 or 47 or 48 or 49 or 50 or 51 or 52 or 53 or 54 or 55 or 56 or 57 or 58 or 59 or 60 or 61 or 62 or 63 or 64 or 65 or 66 or 67 or 68 or 69 or 70 or 71 or 72 or 73 or 74 or 75 or 76 or 77 or 78 or 79 or 80 or 81 or 82 or 83 or 84 or 85 or 86 or 87 or 88
92	89 and 90 and 91

3. Web of Science Search Strategy

edentulous OR edentate OR edentul* OR "total tooth loss" AND "socioeconomic factors" OR "social class" OR income OR salaries OR "educational status" OR productivity OR "nutritional status" OR "dietary intake" OR eating OR anthropometry OR "body fat distribution" OR "body mass index" OR "body size" OR "body surface area" OR "waist-hip ratio" OR "socioeconomic status" OR wealth OR "subjective social status" OR "educational level" OR "educational attainment" OR "education status" OR employment or employability OR productivity OR "handgrip strength" OR "handgrip weakness" OR slowness OR exhaustion OR "low energy" OR "low physical activity" OR "unintentional weight loss" OR frailty OR disability OR mortality OR "premature mortality" OR death OR "Premature death" OR "Excess mortality" OR "excess death" OR "health inequalities" OR "oral health related quality of life" AND adult* OR "middle-aged" OR elderly AND "high income countr*" OR "high income region" OR "developed countr*" OR "developed nation" OR "developed region" OR "wealthy nation" OR "wealthy region" OR "wealthy countr*" OR "rich nation" OR "rich countr*" OR "rich region" OR "middle income countr*" OR "middle income region" OR "middle income nation" OR "low income nation" OR "low income region" OR "low income countr*" OR "developing countr*" OR "developing nation" OR "developing region" OR "third world countr*" OR "third world region" OR "third world nation" OR "poor nation" OR "poor countr*" OR "poor region" OR "deprived nation" OR "deprived region" OR "deprived countr*" OR "underserved nation" OR "underserved countr*" OR "underserved region" OR "transitional region" OR "transitional countr*" OR "transitional nation" OR "low and middle income region" OR "low and middle income nation" OR "low and middle income countr*" OR LMIC OR LAMIC OR globalisation OR global

4. **Scopus Search Strategy**

edentulous OR edentate OR edentul* OR "total tooth loss" AND "socioeconomic factors" OR "social class" OR income OR salaries OR "educational status" OR productivity OR "nutritional status" OR "dietary intake" OR eating OR anthropometry OR "body fat distribution" OR "body mass index" OR "body size" OR "body surface area" OR "waist-hip ratio" OR "socioeconomic status" OR wealth OR "subjective social status" OR "educational level" OR "educational attainment" OR "education status" OR employment or employability OR productivity OR "handgrip strength" OR "handgrip weakness" OR slowness OR exhaustion OR "low energy" OR "low physical activity" OR "unintentional weight loss" OR frailty OR disability OR mortality OR "premature mortality" OR death OR "Premature death" OR "Excess mortality" OR "excess death" OR "health inequalities" OR "oral health related quality of life" AND adult* OR "middle-aged" OR elderly AND "high income countr*" OR "high

income region" OR "developed countr*" OR "developed nation" OR "developed region" OR "wealthy nation" OR "wealthy region" OR "wealthy countr*" OR "rich nation" OR "rich countr*" OR "rich region" OR "middle income countr*" OR "middle income region" OR "middle income nation" OR "low income nation" OR "low income region" OR "low income countr*" OR "developing countr*" OR "developing nation" OR "developing region" OR "third world countr*" OR "third world region" OR "third world nation" OR "poor nation" OR "poor countr*" OR "poor region" OR "deprived nation" OR "deprived region" OR "deprived countr*" OR "underserved nation" OR "underserved countr*" OR "underserved region" OR "transitional region" OR "transitional countr*" OR "transitional nation" OR "low and middle income region" OR "low and middle income nation" OR "low and middle income countr*" OR LMIC OR LAMIC OR globalisation OR global

5. **CENTRAL Search Strategy**

ID	Search	Hits
#1	edentulous OR edentate OR edentul* OR "total tooth loss"	2194
#2	"socioeconomic factors" OR "social class" OR income OR salaries OR "educational status" OR productivity OR "nutritional status" OR "dietary intake" OR eating OR anthropometry OR "body fat distribution" OR "body mass index" OR "body size" OR "body surface area" OR "waist-hip ratio" OR "socioeconomic status" OR wealth OR "subjective social status" OR "educational level" OR "educational attainment" OR "education status" OR employment or employability OR productivity OR "handgrip strength" OR "handgrip weakness" OR slowness OR exhaustion OR "low energy" OR "low physical activity" OR "unintentional weight loss" OR frailty OR disability OR mortality OR "premature mortality" OR death OR "Premature death" OR "Excess mortality" OR "excess death" OR "health inequalities" OR "oral health related quality of life"	286974
#3	"high income countr*" or "high income region" or "developed countr*" or "developed nation" or "developed region" or "wealthy nation" or "wealthy region" or "wealthy countr*" or "rich nation" or "rich countr*" or "rich region" or "middle income countr*" or "middle income region" or "middle income nation" or "low income nation" or "low income region" or "low income countr*" or "developing countr*" or "developing nation" or "developing region" or "third world countr*" or "third world region" or "third world nation" or "poor nation" or "poor countr*" or "poor region" or "deprived nation" or "deprived region" or "deprived countr*" or "underserved nation" or "underserved countr*" or "underserved region" or "transitional region" or "transitional countr*" or "transitional nation" or "low and middle income region" OR "low and middle income nation" or "low and middle income countr*" OR LMIC OR LAMIC or globalisation or global	58794
#4	adult* OR "middle-aged" OR elderly	854508

#5	#1 AND #2 AND #3 AND #4	15
