# Supplementary material for: Impact of edentulism on community-dwelling adults in low-income, middle-income and high-income countries: a systematic review
Source: BMJ Open. 2024 Dec 4;14(12):e085479. doi: 10.1136/bmjopen-2024-085479 (PMC11624734; doi:10.1136/bmjopen-2024-085479)
Supplement: online supplemental file 2 [file bmjopen-14-12-s002.pdf]

Appendix 2: Eligibility Criteria

Inclusion Criteria	Human epidemiological studies of community dwelling completely edentulous adults aged 18 and over residing in low-income, middle-income and high-income countries.<br>This will include experimental studies (randomised controlled trials) and observational studies (cohort studies, case control studies and cross-sectional studies).
Exclusion Criteria	Animal studies.<br>Studies exclusive to partially dentate individuals.<br>Studies exclusive to dentate individuals.<br>Studies exclusive to treated edentulous individuals (dentures, implants).<br>Studies of children and adolescents aged 17 and under.<br>Studies of exclusively non-community dwelling individuals.<br>Non- primary research articles including reviews and systematic reviews.
Restrictions	No language restrictions.<br>No journal category restrictions.
Electronic Database Sources	MEDLINE,<br>EMBASE,<br>Web of Science,<br>Cochrane Library,<br>Scopus.
Final Search Date	21st March 2023
