# Supplementary material for: Impact of edentulism on community-dwelling adults in low-income, middle-income and high-income countries: a systematic review
Source: BMJ Open. 2024 Dec 4;14(12):e085479. doi: 10.1136/bmjopen-2024-085479 (PMC11624734; doi:10.1136/bmjopen-2024-085479)
Supplement: online supplemental file 3 [file bmjopen-14-12-s003.pdf]

Appendix 3: Data Extraction Template

General Information	Article Title
	Authors
	Publication Year
	Country
	Country income status (at time of publication)
	Author contact details
Method	Study design
	Multi- morbidity study yes/no
	Primary or secondary data collection
Participants	Setting
	Total number of participants
	Mean age and Standard Deviation
	Age range
	Sex distribution
	Relevant inclusion criteria
	Relevant exclusion criteria
Characteristics	Percentage of edentulous participants
	Percentage of denture wearers (if stated)
	Percentage of implant treated participants (if stated)
	Comparators used in the study
Outcomes	Primary Outcome of Study: Nutrition/ Mental Health/ Frailty/ Health Bheaviour/ General Health/ Quality of Life/ Mortality/Sleep
	Secondary Outcome (if applicable)
	Evidence of Effectiveness Yes/ No
	Evidence Summary
	Other Subjective Findings
