# Supplementary material for: Impact of edentulism on community-dwelling adults in low-income, middle-income and high-income countries: a systematic review
Source: BMJ Open. 2024 Dec 4;14(12):e085479. doi: 10.1136/bmjopen-2024-085479 (PMC11624734; doi:10.1136/bmjopen-2024-085479)
Supplement: online supplemental file 4 [file bmjopen-14-12-s004.pdf]

Appendix 4: QualSyst Tool Criteria

Criteria	Assessment
1	Question/ objective sufficiently described?
2	Study design evident and appropriate?
3	Method of subject/ comparison group selection or source of information/ input variables described and appropriate?
4	Subject (and comparison group if applicable) characteristics sufficiently described
5	If interventional and random allocation was possible, was it described?
6	If interventional and blinding of investigators was possible, was it described?
7	If interventional and blinding of subjects was possible, was it described?
8	Outcome and (if applicable) exposure measure(s) well defined and robust to minimize measurement/ misclassification bias? Means of assessment reported?
9	Sample size appropriate?
10	Analytic methods described/ justified and appropriate?
11	Some evidence of variance is reported for the main results?
12	Controlled for confounding?
13	Results reported in sufficient detail?
14	Conclusions supported by the results?
