# Supplementary material for: Impact of edentulism on community-dwelling adults in low-income, middle-income and high-income countries: a systematic review
Source: BMJ Open. 2024 Dec 4;14(12):e085479. doi: 10.1136/bmjopen-2024-085479 (PMC11624734; doi:10.1136/bmjopen-2024-085479)
Supplement: online supplemental file 5 [file bmjopen-14-12-s005.pdf]

Appendix 5: Title Excluded Studies

Number	Article Title	First Author	Reason for Exclusion	Additional Notes
1	"Association of tooth loss with morbidity and mortality by diabetes status in older adults: a systematic review"	Raju	Wrong Study Design	Systematic Review
2	"Is Immunosuppression a Risk Factor for Development of Peri-implantitis? A Retrospective Cohort Analysis"	Dazen	Not relevant	Implant Study- Peri implantitis
3	"Is Bone Grafting during Implant Placement a Protective Factor against the Development of Peri-implantitis? A Retrospective Cohort Analysis"	Rekawek	Not relevant	Implant Study- Peri implantitis
4	"Periodontitis as a risk for oral cancer: a case-control study"	Komlos	Not relevant	Oral cancer study
5	"Development of patient-centric eating advice for complete denture wearers"	Al-Sultani	Not relevant	Patient advice for denture wear
6	"Funding assistance and global productivity in the field of implant overdentures: A bibliometric analysis of 35 years"	Borges	Not relevant	Funding for implant over dentures
7	"Evaluation of patient satisfaction and masticatory performance in mandible single implant-assisted overdenture"	Mittal	Not relevant	Patient satisfaction study implants

Number	Article Title	First Author	Reason for Exclusion	Additional Notes
8	"Evaluation of DO Vent YT intraoral device to facilitate ventilation with facial mask in patients with difficult ventilation risk"	Tenia	Not relevant	Anaesthetic
9	"Age-related Oral Changes and Their Impact on Oral Health-related Quality of Life among Frail Elderly Population: A Review"	Naik	Wrong Study Design	Review
10	"Combined intraoral and lateral temporal approach for palatal malignancies with temporalis muscle reconstruction"	Browne, Holland	Not relevant	Oral cancer study
11	"Evaluation of quality of life and patient satisfaction between denture wearers using conventional followed by bio functional prosthetic system (Bps) dentures"	Kasabwala	Wrong population	Denture Wearers
12	"How peoples' ratings of dental implant treatment change over time?"	Machuca	Not relevant	Implant study- satisfaction with implants
13	"Evaluation of the clinical efficacy of implant-supported overdenture with edentulous mandible in elderly patients"	Huang, Lin.	Not relevant	Implant study- clinical effectiveness
14	"A randomized controlled trial on the efficiency of free-handed, pilot-drill guided and fully guided implant surgery in partially edentulous patients"	Younes	Not relevant	Implant study- surgical technique

Number	Article Title	First Author	Reason for Exclusion	Additional Notes
15	“Teaching Complete Denture Procedures to Dental Students by Conventional or Simplified Methods: A Randomized Clinical Trial”	de Villa Camargos	Not relevant	Prosthetics-education for dental students
16	“The psychometric properties of a new oral health illness perception measure for adults aged 62 years and older”	Nelson	Not relevant	Development of disease measure
17	“Chlorhexidine oral topical application for critical patients: Savior or killer?”	Bellissimo-	Not relevant	ICU patient management
18	“Sociodemographic determinants of edentulism in the elderly population: A systematic review and meta-analysis”	Roberto	Wrong study design	Systematic Review
19	“The impact of demographic, health-related and social factors on dental services utilization: Systematic review and meta-analysis”	Reda	Wrong study design	Systematic Review
20	“Group 3 ITI Consensus Report: Patient-reported outcome measures associated with implant dentistry”	Feine	Not relevant	Development of disease measure
21	“Relationship of nutritional status and oral health in elderly: Systematic review with meta-analysis”	Toniazzo	Wrong Study Design	Systematic Review
22	“Expectation and fulfillment of partially edentulous patients	Dhamini	Wrong population	Participants are partially dentate

Number	Article Title	First Author	Reason for Exclusion	Additional Notes
	receiving removable partial dentures in a south Indian dental college”			
23	“The influence of intraoral devices on static performance in golf athletes-pilot study”	Santos	Not relevant	Sports medicine
24	“Reliability and Validity of a Nepalese Version of the Oral Health Impact Profile for Edentulous Subjects”	Shrestha	Not relevant	Development of disease measure
25	“Associations between tooth loss, with or without dental prostheses, and malnutrition risk in older adults: A systematic review”	Zelig	Wrong Study Design	Systematic Review
26	“Prevalence of periodontal disease in the general population of India-A systematic review”	Shewale	Wrong Study Design	Systematic Review
27	“Relationship between oral health and cognition in older patients admitted in a medical ward of a general hospital”	Duque	Wrong population	Non-community dwelling
28	“Skill-mix in preventive dental practice--will it help address need in the future?”	Brocklehurst, Macey	Not relevant	Dental services-skill mix
29	“A new species of Hatchet-faced Treefrog Sphaenorhynchus Tschudi (Anura: Hylidae) from Quadrilatero Ferrifero, Minas Gerais, south-eastern Brazil”	Araujo-Vieira	Not relevant	Non-human study

Number	Article Title	First Author	Reason for Exclusion	Additional Notes
30	“Determinants of protein-energy malnutrition in community-dwelling older adults: A systematic review of observational studies”	van der Pols-Vijlbrief	Wrong Study Design	Systematic Review
31	“Determinants of undernutrition among community dwelling older adults: A systematic literature review”	Van Der Pols-Vijlbrief	Wrong Study Design	Literature Review
32	“Oral health of patients in an acute geriatric ward in a teaching hospital in Kuala Lumpur, Malaysia”	Long	Wrong population	Not community dwelling- prevalence study also
33	“A structural equation model relating oral condition, denture quality, chewing ability, satisfaction, and oral health-related quality of life in complete denture wearers”	Yamaga	Wrong population	Complete denture wearers
34	“Dental treatments received by elderly in two economically different countries”	Vehkalahti	Not relevant	Dental services- treatments available
35	“A challenging case of field cancerization”	Bektas-Kayhan	Not relevant	Oral cancer study
36	“A multi-centered epidemiological study evaluating the reliability of the treatment difficulty indices developed by the Japan Prosthodontic Society”	Kuboki	Not relevant	Disease measure study

Number	Article Title	First Author	Reason for Exclusion	Additional Notes
37	"Derivation of the short form of the Oral Health Impact Profile in Spanish (OHIP-EE-14)"	Castrejon-Perez, Borges-Yanez	Not relevant	Disease measure study
38	"Perceptions and preferences on denture marking in an Indian sample"	Acharya	Not relevant	Preference on denture marking study
39	"Assessing oral health promotion determinants in active Greek elderly"	Naka, Anastassiad ou	Not relevant	Health promotion study
40	"Impact of rehabilitation with metal-ceramic restorations on oral health-related quality of life"	da Silva	Wrong population	Dentate-restorative study
41	"Development of a patient-based questionnaire about emotional and social issues related to eating with dentures"	Kelly	Not relevant	Development of questionnaire study
42	"Recovering the personal identity of an elderly patient with cleft lip: A case report"	Khalil	Not relevant	Cleft lip study
43	"Kennedy Classification and Treatment Options: A Study of Partially Edentulous Patients Being Treated in a Specialized Prosthetic Clinic"	Charyeva	Wrong population	Dentate patients-partial denture
44	"A novel decision-making process for tooth retention or extraction"	Avila	Wrong population	Dentate patients-restorative

Number	Article Title	First Author	Reason for Exclusion	Additional Notes
45	“Change in oral health status among the institutionalized Norwegian elderly over a period of 16 years”	Samson	Wrong outcome	Prevalence study
46	“The global burden of oral diseases and risks to oral health”	Petersen	Wrong outcome	Prevalence study
47	“Developing short-form measures of oral health-related quality of life”	Locker, Allen	Not relevant	Disease measure study
48	“A comparison of the validity of generic- and disease-specific measures in the assessment of oral health-related quality of life”	Allen	Not relevant	Disease measure study
49	“Dental effects of diet and coca-leaf chewing on two prehistoric cultures of northern Chile”	Langsjoen	Not relevant	Dental history-prehistoric
50	“Caries prevention. A continued need worldwide”	Blinkhorn, Davies	Wrong population	Economic burden of periodontitis in the United States and Europe: An updated estimation
51	“A utility analysis of dental implants”	Jacobson	Not relevant	Implant study-utility
52	“Hospital-based pilot study on partially dentate and edentate patients to evaluate disparity between prosthodontic treatment demand and need: a cross-sectional sociodemographic study”	Singh	Wrong outcome	Prosthetics- need and demand

Number	Article Title	First Author	Reason for Exclusion	Additional Notes
53	“Economic burden of periodontitis in the United States and Europe: An updated estimation”	Botelho	Wrong population	Dentate- perio economics
54	“Critical assessment on unmet oral health needs and oral health-related quality of life among old age home inhabitants in Karnataka, India”	Roma	Wrong population	Not community dwelling
55	“Age-sex specific and sequela-specific disability-adjusted life years (DALYs) due to dental caries preventable through water fluoridation: An assessment at the national and subnational levels in Iran, 2016”	Abtahi	Wrong population	Dentate- caries
56	“Determinants of not availing oral prosthesis in low income country- Pakistan: A cross sectional survey in Lahore population”	Rasul	Wrong outcome	Prosthetics- need and demand
57	“Differentiation of face and auricular shape resulting from diabetes and hypertension in the elderly”	Nunes	Not relevant	Impact on face shape
58	“Social gradient in caries experience of Belgian adults 2010”	Lambert	Wrong population	Caries
59	“Oral health in nursing home residents with different cognitive statuses”	Chen	Wrong population	Not community dwelling
60	“Prevalence of partial edentulousness and treatment needs in rural population of South India”	Ashraf	Wrong outcome	Prevalence study

Number	Article Title	First Author	Reason for Exclusion	Additional Notes
61	"Geriatric periodontology: how the need to care for the aging population can influence the future of the dental profession"	Lamster	Not relevant	Dental services- skill mix
62	"Are people who still have their natural teeth willing to pay for mandibular two-implant overdentures?"	Srivastava	Not relevant	Prosthetics- need and demand
63	"Analysis of the association of IL1B(C-511T) polymorphism with dental implant loss and the clusterization phenomenon"	Dirschnabel	Not relevant	Implant study- clinical effectiveness
64	"A short report on tooth replacement in an older suburban population in Nigeria"	Ibiyemi, Lawal	Wrong outcome	Prevalence study
65	"Prevention and management of periodontal diseases and dental caries in the older adults"	Al-Nasser, Lamster,	Wrong population	Dentate- perio and caries
66	"Oral health in the elderly patient and its impact on general well-being: a nonsystematic review"	Gil-Montoya	Wrong study design	Review
67	"The impact of oral rehabilitation coupled with healthy dietary advice on the nutritional status of adults: A systematic review and meta-analysis"	McGowan	Wrong Study Design	Systematic Review
68	"Factors Associated With Becoming Edentulous in the US Health and Retirement Study"	Weintraub	Wrong outcome	Edentulism is outcome not exposure

Number	Article Title	First Author	Reason for Exclusion	Additional Notes
69	"Prevalence of tooth mortality among adults in India: A systematic review and meta-analysis"	Janakiram	Wrong Study Design	Systematic Review
70	"The burden of tooth loss in Italian elderly population living in nursing homes"	Cocco	Wrong population	Non-community dwelling
71	"Oral health status of adults in North Carolina assisted living facilities"	Stephens, White	Wrong population	Non-community dwelling
72	"The Causal Effect of Education on Tooth Loss: Evidence From United Kingdom Schooling Reforms"	Matsuyama	Wrong outcome	Edentulism is outcome not exposure
73	"Periodontitis and Italians (2016-2020): need for clinical guidelines to perform effective therapy"	Raspini	Wrong population	Dentate- perio
74	"Final-impression techniques and materials for making complete and removable partial dentures"	Jayaraman	Not relevant	Prosthetics-technique
75	"Interventions for the management of mandibular fractures"	Nasser	Not relevant	Trauma
76	"Interventions for replacing missing teeth: management of soft tissues for dental implants"	Esposito	Not relevant	Implant study-surgical technique
77	"Oral care measures for preventing nursing home-acquired pneumonia"	Liu	Not relevant	Dental services-OHI and diet
78	"One-to-one dietary interventions undertaken in a dental setting to change dietary behaviour"	Harris	Not relevant	Dental services-OHI and diet

Number	Article Title	First Author	Reason for Exclusion	Additional Notes
79	"Interventions for replacing missing teeth: attachment systems for implant overdentures in edentulous jaws"	Payne	Not relevant	Implants- techniques
80	"Oral hygiene care for critically ill patients to prevent ventilator-associated pneumonia"	Zhao	Not relevant	Dental services- OHI and diet
81	"Interventions for replacing missing teeth: hyperbaric oxygen therapy for irradiated patients who require dental implants"	Esposito, Worthington	Not relevant	Implants- techniques
82	"Interventions for treating bisphosphonate-related osteonecrosis of the jaw (BRONJ)"	Rollason	Not relevant	In relation to bisphosphonate related osteonecrosis
83	"Fluoride varnishes for preventing dental caries in children and adolescents"	Marinho	Wrong population	Caries<br>Children
84	"Training health professionals in smoking cessation"	Carson	Not relevant	Education for professionals
85	"Adjunctive systemic antimicrobials for the non-surgical treatment of periodontitis"	Khattri	Wrong population	Dentate- perio
86	"Tooth Loss and Nutritional Status in Older Adults: A Systematic Review and Meta-analysis"	Zelig	Wrong study design	Systematic Review

**Number**	**Article Title**	**First Author**	**Reason for Exclusion**	**Additional Notes**
87	"Common risk factors and edentulism in adults, aged 50 years and over, in China, Ghana, India and South Africa: results from the WHO Study on global AGEing and adult health (SAGE)"	Kailembo	Wrong outcome	Edentulism is outcome not exposure
88	"Partial edentulism and unmet prosthetic needs amongst young adult Nigeria"	Akinboboye	Wrong population	Dentate patients- partial denture
89	"The future of complete dentures in oral rehabilitation. A critical review"	Carlsson, Omar	Wrong study design	Review
90	"Attitudes and perception of Nigerian dentists about shortened dental arch therapy (SDAT)"	Arigbede	Wrong population	Dentate patients- partial denture
91	"Dental caries experience of British children in an international context"	Downer	Wrong population	Caries<br>Children
92	"Reappraising prosthodontic treatment goals for older, partially dentate people: Part II. Case for a sustainable dentition?"	Omar, Ridwaan	Wrong population	Dentate patients- partial denture
93	"Does case severity make a difference to clinical improvement following complete denture treatment"	Kurushima	Wrong study design	Clinical outcomes
94	"Overdentures on Implants for Better Quality of Life Among the Fully Edentulous Patients- Case Reports"	Nikolovska	Wrong study design	Case reports

Number	Article Title	First Author	Reason for Exclusion	Additional Notes
95	"Assessment of Quality of Life in Head-and-Neck Oncologic Patients with Intraoral Soft-Tissue Defects Reconstructed with Buccinator Myomucosal Flap"	Agea Martínez	Not relevant	Cancer treatment
96	"Global prevalence of edentulism and dental caries in middle-aged and elderly persons: A systematic review and meta-analysis"	Borg-Bartolo	Wrong study design	Systematic Review
97	"Incidence of edentulism among older adults using the Korean National Health Insurance Service database, 2013-2018"	Go	Wrong outcome	Incidence study
98	"Prediction Model Development and Validation of 12-Year Incident Edentulism of Older Adults in the United States"	Preisser	Not relevant	Prediction modelling
99	"Tolerance and Benefits of Mandibular Advanced Device for Snoring and Sleep Apnea in Oropharyngeal Cancer"	Cochrane Library Trial Registry Record	Not relevant	Snoring and sleep apnea
