# Supplementary material for: Impact of edentulism on community-dwelling adults in low-income, middle-income and high-income countries: a systematic review
Source: BMJ Open. 2024 Dec 4;14(12):e085479. doi: 10.1136/bmjopen-2024-085479 (PMC11624734; doi:10.1136/bmjopen-2024-085479)
Supplement: online supplemental file 6 [file bmjopen-14-12-s006.pdf]

Appendix 6: Abstract Excluded Studies

Number	Title	First Author	Exclusion reason	Additional Notes
1	"Perceived status and care practices among complete denture wearers"	Sharma	Wrong population	Exclusive to complete denture wearers
2	"Nutritional assessment and associated factors in the elderly: a population-based cross-sectional study"	Stoffel	Wrong population	Non-community dwelling
3	"Prevalence, incidence, and years-lived with disability due to oral disorders in Brazil: an analysis of the Global Burden of Disease Study 2019"	Hugo	Wrong outcome	Prevalence study
4	"Association between social capital and oral health among adults aged 50 years and over in China: a cross-sectional study"	Li	Wrong outcome	Edentulism is outcome not exposure

Number	Title	First Author	Exclusion reason	Additional Notes
5	"Oral Health of Older Patients in Dental Practice: An Exploratory Study"	Bots-VantSpijker	Wrong outcome	Prevalence study
6	"Oral health status of patients infected with human immunodeficiency virus and related factors, Iran: a cross-sectional study"	Shaghaghian	Wrong outcome	Edentulism risk factor study not consequence
7	"Association between diabetes and edentulism and their joint effects on health status in 40 low and middle-income countries"	Jacob	Wrong outcome	Edentulism is outcome not exposure
8	"Oral health status and associated lifestyle behaviors in a sample of Iranian adults: an exploratory household survey"	Jahangiry	Wrong outcome	Prevalence study

Number	Title	First Author	Exclusion reason	Additional Notes
9	"Health insurance and education: major contributors to oral health inequalities in Colombia	Guarnizo-Herreno	Wrong outcome	Edentulism is outcome not exposure
10	"Assessment of oral health status among uncontrolled diabetic mellitus patients in Tunisia	Sebai	Wrong outcome	Prevalence study
11	"Socioeconomic inequalities in adult oral health across different ethnic groups in England"	Delgado-Angulo	Wrong outcome	Edentulism is outcome not exposure
12	"Racial or ethnic health disparities among older adults in four population groups in South Africa"	Phaswana-Mafuya, Peltzer	Wrong outcome	Prevalence study
13	"Association between tooth loss and hypertension among	Singh A	Wrong outcome	Study on partial tooth loss not total tooth loss

Number	Title	First Author	Exclusion reason	Additional Notes
	a primarily rural middle aged and older Indian adult population”			
14	“Global burden of oral diseases: emerging concepts, management and interplay with systemic health”	Jin	Wrong outcome	Edentulism is outcome not exposure
15	“Comparison of Oral Health Status in Asia: Results for Eight Emerging and Five High Income Countries or Regions and Implications”	Saekel	Wrong outcome	Prevalence study
16	“Oral health in older patients with oropharyngeal dysphagia”	Ortega	Wrong outcome	Prevalence study
17	“Oral health status of patients with psychiatric problem”	Rahman	Wrong population	Dentate

Number	Title	First Author	Exclusion reason	Additional Notes
18	"Goals for oral health in Tunisia 2020"	Maatouk	Not relevant	Health policy
19	"Obstructive airway disease and edentulism in the atherosclerosis risk in communities (ARIC) study"	Offenbacher	Wrong outcome	Edentulism is outcome not exposure
20	"Association between self-assessment of complete dentures and oral health-related quality of life	Komagamine	Wrong population	Exclusive to complete denture wearers
21	"Validation of the oral health impact profile (OHIP-20sp) for Spanish edentulous patients	Montero	Not relevant	Disease measure study
22	"Perceived sleep quality among edentulous elders"	Emami	Wrong population	Exclusive to complete denture wearers

Number	Title	First Author	Exclusion reason	Additional Notes
23	“Epidemiological survey on edentulousness”	Reddy	Wrong outcome	Edentulism is outcome not exposure
24	“Oral health-related quality of life and oral status in a German working population”	Walter	Wrong population	Dentate
25	“Investigation on how renewal of complete dentures impact on dietary and nutrient adequacy in edentulous patients”	Gunji	Wrong population	Exclusive to complete denture wearers
26	“Association between Oral Health Impact Profile and General Health scores for patients seeking dental implants”	Smith	Wrong population	Patients seeking implants may not be reflective of all edentulous patients
27	“Factors associated with oral health perception in older Brazilians”	Nunes, Abegg	Wrong outcome	Edentulism is outcome not exposure

Number	Title	First Author	Exclusion reason	Additional Notes
28	“Quality of life and masticatory function in denture wearers”	Koshino	Wrong population	Exclusive to denture wearers
39	“Correlation between quality of life and denture satisfaction in elderly complete denture wearers”	Yoshida	Wrong population	Exclusive to complete denture wearers
30	“The impact of the demographics of aging and the edentulous condition on dental care services”	Thompson, Kreise	Wrong outcome	Prevalence study
31	“Oral health status and traditions in the Philippines”	Gamboa	Wrong outcome	Prevalence study
32	“Oral health status in the Pars Cohort Study”	Ghazimoghadam	Wrong outcome	Prevalence study
33	“Rural-urban health disparities among older adults in South Africa”	Peltzer	Wrong outcome	Prevalence study

**Number**	**Title**	**First Author**	**Exclusion reason**	**Additional Notes**
34	"Evaluation of relationship between nutritional status and oral health related quality of life in complete denture wearers"	Banerjee	Wrong population	Exclusive to complete denture wearers
35	"Midlife Cognitive Ability, Education, and Tooth Loss in Older Danes"	Bachkati	Wrong outcome	Edentulism is outcome not exposure
36	"Social inequalities in adult oral health in 40 low- and middle-income countries"	Bhandari	Wrong outcome	Edentulism is outcome not exposure
37	"Socioeconomic status, health behaviours and oral health in adult urban population of Krakow"	Łysek	Wrong outcome	Edentulism is outcome not exposure
38	"Burden of oral diseases in Iran, 1990-2010: Findings	Shoaee	Study design	Systematic Review

Number	Title	First Author	Exclusion reason	Additional Notes
	from the global burden of disease study 2010”			
39	“Global burden of oral conditions in 1990-2010: A systematic analysis”	Marcenes	Wrong outcome	Prevalence study
40	“Assessment of predictors of global self-ratings of oral health among Korean adults aged 18-95 years”	Kim	Wrong outcome	Study on influence of age and GSROH
41	“Determining the minimally important difference for the Oral Health Impact Profile-20”	Allen	Wrong population	All participants partially dentate
42	“Impact of new prostheses on the oral health related quality of life of edentulous patient”	Veyrune	Wrong population	Exclusive to complete denture wearers

Number	Title	First Author	Exclusion reason	Additional Notes
43	"The relationship between dental status and health-related quality of life in upper aerodigestive tract cancer patients"	Allison	Wrong population	Exclusive to complete denture wearer
44	"Tooth Mortality in an Adult Rural Population in Kenya"	Manji	Wrong outcome	Prevalence study
45	"Socioeconomic inequality in self-reported unmet need for oral health services in adults aged 50 years and over in China, Ghana, and India"	Kailembo	Wrong outcome	Dental services
46	"China's Oral Care System in Transition: Lessons to be Learned from Germany"	Saekel	Wrong outcome	Dental services

Number	Title	First Author	Exclusion reason	Additional Notes
47	"Is tooth loss important when evaluating perceived general health? Findings from a nationally representative study of Costa Rican adults"	Barboza-Soli	Wrong outcome	Dental services<br>Disease measure study
48	"Wider Dental Care Coverage Associated with Lower Oral Health Inequalities: A Comparison Study between Japan and England"	Ito	Wrong outcome	Dental services
49	"Edentulism and associated factors among community-dwelling middle-aged and elderly adults in China"	Ren	Wrong outcome	Prevalence study
50	"The nocturnal use of complete dentures and sleep stability in edentulous elders"	Emami	Wrong population	Exclusive to complete denture wearers

Number	Title	First Author	Exclusion reason	Additional Notes
51	“The impact of dental status on perceived ability to eat certain foods and nutrient intakes in older adults: cross-sectional analysis of the UK National Diet and Nutrition Survey 2008-2014”	Watson	Wrong population	Exclusive to complete denture wearers
52	“Social stratification and tooth loss among middle-aged and older Americans from 1988 to 2004”	Wu	Wrong outcome	Edentulism is outcome not exposure
53	“Impact of adopting different socioeconomic indicators in older adults' oral health research”	do Amaral, OL	Wrong outcome	Edentulism is outcome not exposure
54	“Association between Underweight and Edentulism among Older (50+) Men and Women in India”	Selvamani, Singh	Wrong outcome	Edentulism is outcome not exposure

**Number**	**Title**	**First Author**	**Exclusion reason**	**Additional Notes**
55	"Individual- and community-level social gradients of edentulousness"	Ito	Wrong outcome	Edentulism is outcome not exposure
56	"Geriatric oral health predicaments in New Delhi, India"	Singh	Wrong outcome	Dental services
57	"Cognitive Decline and Oral Health in Middle-aged Adults in the ARIC Study"	Naorungroj	Wrong outcome	Edentulism is outcome not exposure
58	"The impact of having natural teeth on the QoL of frail dentulous older people. A qualitative study"	Niesten	Wrong population	Dentate
59	"Patient-centred rehabilitation of edentulism with an optimal number of implants"	Anonymous	Wrong outcome	Implant study

Number	Title	First Author	Exclusion reason	Additional Notes
60	“Life course socioeconomic position indicators and tooth loss in Korean adults”	Han, Khang,	Wrong outcome	Edentulism is outcome not exposure
61	“Geriatric oral health issues in the United Kingdom”	Walls, Steele	Wrong outcome	Prevalence study
62	“Tooth loss and associated factors in elders: results from a national survey in Uruguay”	Laguzzi	Wrong outcome	Prevalence study
63	“Oral health diseases among the older people: a general health perspective”	Liu	Study design	Review
64	“Oral health educational interventions for nursing home staff and residents”	Albrecht	Study design	Review
65	“Patterns of oral disease in adults with chronic kidney disease treated with hemodialysis”	Palmer	Wrong outcome	Edentulism is outcome not exposure

Number	Title	First Author	Exclusion reason	Additional Notes
66	“Tooth loss among older adults according to poverty status in the United States from 1999 through 2004 and 2009 through 2014”	Dye	Wrong outcome	Prevalence study
67	“Association between tooth loss, chronic conditions, and common risk factors-Results from the 2019 Brazilian Health Survey”	de Medeiros	Wrong outcome	Edentulism is outcome not exposure
68	“Oral health status of nursing staff in Ilembula, Wanging'ombe District, Njombe region, Tanzania: a cross-sectional study”	Bensel	Wrong outcome	Prevalence study
69	“Oral health parameters in the regional study among young seniors in an urban area of Wroclaw”	Konopka	Wrong outcome<br>Wrong population	Caries and Perio<br>Dentate

Number	Title	First Author	Exclusion reason	Additional Notes
70	"Socio-demographic factors and edentulism: the Nigerian experience"	Esan	Wrong outcome	Edentulism is outcome not exposure
71	"Global, Regional, and National Levels and Trends in Burden of Oral Conditions from 1990 to 2017: A Systematic Analysis for the Global Burden of Disease 2017 Study"	Bernabe	Wrong outcome	Prevalence study
72	"Prevalence of loss of all teeth (edentulism) and associated factors in older adults in China, Ghana, India, Mexico, Russia and South Africa"	Peltzer	Wrong outcome	Edentulism is outcome not exposure
73	"Socioeconomic factors and complete edentulism in north karnataka population"	Nagaraj	Wrong outcome	Prevalence study

Number	Title	First Author	Exclusion reason	Additional Notes
74	“The global burden of periodontal disease: towards integration with chronic disease prevention and control”	Petersen,	Wrong outcome	Prevalence study
75	“Population ageing and dental care”	Harford	Wrong outcome	Prevalence study
76	“The number of children, use of oral contraceptives and menopausal status in relation to the number of remaining teeth and the periodontal bone height. A population study of women in Gothenburg, Sweden”	Halling, Bengtsson	Wrong outcome	Edentulism is outcome not exposure
77	“The nutritional effects of tooth loss”	Geissler, Bates	Study design	Review

Number	Title	First Author	Exclusion reason	Additional Notes
78	Contextual and individual factors associated with self-reported tooth loss among adults and elderly residents in rural riverside areas: A cross-sectional household-based survey.	de Souza VGL	Wrong outcome	Edentulism is outcome not exposure
79	Association between clinical oral health status and perceived oral health in different age groups	Fahim	Wrong population	Caries and Perio<br>Dentate
80	Multimorbidity, depression with anxiety symptoms, and decrements in health in 47 low- and middle-income countries	Felez-Nobrega	Not relevant	Edentulism not mentioned
81	Dental Care for Older Adults	Leung Chu	Wrong study design	Communication article

Number	Title	First Author	Exclusion reason	Additional Notes
82	Spatiotemporal trends of disease burden of edentulism from 1990 to 2019: A global, regional, and national analysis.	Li	Wrong outcome	Prevalence study
83	Using machine learning algorithms to investigate factors associated with complete edentulism among older adults in the United States	Oladayo	Wrong outcome	Edentulism is outcome not exposure
84	Oral Health, Diet, and Frailty at Baseline of the Canadian Longitudinal Study on Aging	Bassim	Not relevant	Study related to poor oral health, not specifically edentulism
85	A 2-year longitudinal study of the relationship between masticatory function and progression to frailty or pre-frailty among community-	Horibe	Wrong population	Dentate patients. Concerned with chewing ability rather than edentulism

Number	Title	First Author	Exclusion reason	Additional Notes
	dwelling Japanese aged 65 and older.			
86	Relationships between perceived chewing ability and muscle strength of the body among the elderly	Moriy	Wrong population	Not specific to edentulous patients
87	Relationships between Geriatric Oral Health Assessment Index scores and general physical status in community-dwelling older adults	Moriya	Wrong population	Not specific to edentulous patients
88	Relationships between oral conditions and physical performance in a rural elderly population in Japan	Moriya	Wrong population	Self-assessed masticatory ability was assessed but the pattern of occlusal pairs was not- not clear if edentulous patients
89	Influence of dental occlusion on physical fitness decline in a	Okuyama	Wrong population	Dentate patients

Number	Title	First Author	Exclusion reason	Additional Notes
	healthy Japanese elderly population.			
90	Relationship Between Dental Occlusion and Physical Fitness in an Elderly Population	Yamaga	Wrong population	Dentate patients
91	Oral health status and change in handgrip strength over a 5-year period in 80-year-old people	Hamalainen	Wrong population	Dentate patients
92	Periodontal Disease and Weight Loss in Older Adults	Weyant	Wrong population	Dentate patients
93	Oral Disease and 3-Year Incidence of Frailty in Mexican Older Adults	Castrejón-Pérez	Wrong population	Not specific to edentulous patients
94	Oral health conditions and frailty in Mexican community-dwelling elderly: a cross sectional analysis	Castrejón-Pérez	Wrong population	Not specific to edentulous patients
95	Low Serum Micronutrient Concentrations Predict Frailty	Semba	Not relevant	

Number	Title	First Author	Exclusion reason	Additional Notes
	Among Older Women Living in the Community			
96	A 5-year longitudinal study of association of maximum bite force with development of frailty in community-dwelling older adults	Iwasaki	Wrong population	Dentate patients. Concerned with chewing ability rather than edentulism
97	Dentition status and frailty in community-dwelling older adults: A 5-year prospective cohort study	Iwasaki	Wrong population	Dentate patients. Concerned with chewing ability rather than edentulism
98	Association between objectively measured chewing ability and frailty: A cross-sectional study in central Thailand	Iwasaki	Wrong population	Dentate patients. Concerned with chewing ability rather than edentulism
99	Relationship between masticatory function and frailty in community-dwelling Japanese elderly	Horibe	Wrong population	Dentate patients. Concern with chewing ability rather than edentulism

Number	Title	First Author	Exclusion reason	Additional Notes
100	Oral Frailty as a Risk Factor for Physical Frailty and Mortality in Community-Dwelling Elderly	Tanaka	Wrong population	N- edentulous patients not isolated, simply 'poor oral health'
101	Relationship Between Frailty and Oral Function in Community-Dwelling Elderly Adults	Watanabe	Wrong population	Dentate patients. Concern with chewing ability rather than edentulism
102	Association between number of teeth, use of dentures and musculoskeletal frailty among older adults	Lee, Sabbah	Wrong population	Dentate patients
103	Effects of oral environment on frailty: particular relevance of tongue pressure	Satake	Wrong outcome	Edentulous patients not isolated in the analysis
104	Relationship between oral health and Fried's frailty criteria in community-dwelling older persons	Kamdem	Wrong outcome	Edentulous patients not isolated in the analysis
105	Exploring associations between oral health and frailty in	Everaar	Wrong outcome	Edentulous patients not isolated in the analysis

Number	Title	First Author	Exclusion reason	Additional Notes
	community-dwelling older people			
106	Self-rated oral health and frailty index among older Americans	Hakeem	Wrong outcome	Exposure is related to self-rated oral health (very good, good, fair, poor etc) rather than edentulism
107	Association Between Oral Health and Frailty Among American Older Adults.	Hakeem	Wrong population	Dentate
108	Association between oral health and frailty: results from the Korea National Health and Nutrition Examination Survey	Kim	Wrong outcome	Edentulous patients not isolated in the analysis
