# Supplementary material for: Impact of edentulism on community-dwelling adults in low-income, middle-income and high-income countries: a systematic review
Source: BMJ Open. 2024 Dec 4;14(12):e085479. doi: 10.1136/bmjopen-2024-085479 (PMC11624734; doi:10.1136/bmjopen-2024-085479)
Supplement: online supplemental file 7 [file bmjopen-14-12-s007.pdf]

Appendix 7: Full Text Excluded Studies

Number	Study Title	First Author	Exclusion Reason	Extra Comments
1	"Total tooth loss without denture wear is a risk indicator for difficulty eating among older adults with intellectual disabilities"	Mac Giolla Phadraig	Wrong population	No distinction between community dwelling and non-community dwelling. Author contacted but unable to extract community dwelling data
2	"Periodontal disease and risk of cerebrovascular disease: the first national health and nutrition examination survey and its follow-up study"	Wu	Wrong population	Patients with retained roots considered as edentulous-wrong population
3	"Socioeconomic Disadvantage, Chronic Diseases and their Association with Cognitive Functioning of Adults in India: A Multilevel Analysis"	Kumar	Problem with results	No statistical significance for edentulous patients, only for no disease/ one morbidity or multi morbidity
4	"Relationship between oral impacts on daily performance and chewing ability among independent elders residing in Daejeon City, Korea"	Hwang	Not representative	Author stated not representative of the population of elderly patients in Korea

5	“Tooth loss and obstructive sleep apnoea”	Bucca	Wrong population	All patient’s are denture wearers-
6	“A relationship between tooth loss and periodontal disease with increased blood pressure in adults: A population-based study in Iran”	Moghadam	Problem with results	There are conflicting conclusions in the article.
7	“Assessment of oral health related quality of life among completely edentulous patients in Western India by using GOHAI”	Dable	Study design	RCT with no control arm
8	“Association between edentulism and cognition among people aged 50 and over”	Ruan	No access	Unable to access article. Study authors emailed and QUB library requested but unavailable
9	“Association of diabetes and periodontitis with the incidence of frailty in Mexican elderly”	Borges-Yanez, Castrejon-perez	Problem with results	Results for periodontal disease only available.
10	“Associations between active travel and physical multi-morbidity in six low- and middle-income countries among community-dwelling older adults: A cross-sectional study”	Vancampfort	Wrong outcome	Edentulism is outcome not exposure
11	“Clinical scenario and oral health status in stroke patient”	Budin	Problem with results	Statistical analysis is of DMFT rather than edentulism

12	"Impact of Dental Diseases on Quality-Adjusted Life Expectancy in US Adults"	Matsuyama	Problem with results	No information on edentulous patients, only periodontal disease, decayed teeth and missing teeth but it is not specified if that is total tooth loss or partial tooth loss.
13	"Masticatory handicap, socioeconomic status, and chronic conditions among adults"	Chen	No access	Unable to access article. Study authors emailed and QUB library requested but unavailable
14	"Nonfatal stroke and all-cause mortality among community-dwelling older adults living in rural Ecuador: A population-based, prospective study"	Del Brutto	Wrong outcome	Study is primarily concerned with non-fatal stroke being related to increases in all cause mortality. Edentulism included as a confounder only.
15	"Oral health status and associated factors in minority adults with neurocognitive disorders"	Reinhardt	Insufficient information	
16	"Intra-category determinants of global self-rating of oral health among the elderly"	Kim	Problem with results	In analysis, edentulous data has been combined with dentate data

17	“Oral health issues of Spanish adults aged 65 and over”	The Spanish Geriatric Oral Health Research Group*	Wrong population	Article is mainly regarding prevalence of oral health conditions among Spanish adults.. There is a mixture of community dwelling and institutionalised and the distinction among the edentulous patients is not made
18	“Physical multimorbidity and psychosis: Comprehensive cross-sectional analysis including 242,952 people across 48 low- and middle-income countries”	Stubbs	Problem with results	No statistical signfiicance for edentulous patients.
19	“Epidemiology of depression with psychotic experiences and its association with chronic physical conditions in 47 low- and middle-income countries”	Koyanagi	Wrong outcome	Edentulism the outcome rather than exposure
20	“Utilization of Dental Care, Tooth Loss, and Oral Health-Related Quality of Life in Older Adults Visiting Dental Care Centers in Indian Settings”	Nayyan	Problem with results	No confidence intervals, no statistical analysis
21	“Association between Oral Health and Frailty among Older Adults in Madinah, Saudi Arabia: A Cross-Sectional Study”	Hakeem	Wrong population	Did not specify edentulous patients- only greater or less than 21 teeth
22	“Elderly with remaining teeth report less frailty and better quality of life than edentulous elderly: a cross-sectional study”	Hoeksema	Wong population	Exposure was either older people with remaining teeth or with complete dentures/implants- unclear if any non-denture wearing

				edentulous patients were included
