# Supplementary material for: Impact of edentulism on community-dwelling adults in low-income, middle-income and high-income countries: a systematic review
Source: BMJ Open. 2024 Dec 4;14(12):e085479. doi: 10.1136/bmjopen-2024-085479 (PMC11624734; doi:10.1136/bmjopen-2024-085479)
Supplement: online supplemental file 8 [file bmjopen-14-12-s008.pdf]

Appendix 8: Characteristics of Included Studies

Number	First Author	Study Type	Year of Publication	Number of Participants	% of Edentulous Participants	Country Income	Participant ages	Primary Outcome
1	Albani	Cross-sectional	2021	The Newcastle 85+ Study in England (n = 853) and the Tokyo Oldest Old Survey on Total Health (TOOTH; n = 542) random samples of people aged > 85 years	85+ study: 52.8%<br><br>TOOTH study: 29.5%	High-income	> 85 years	Frailty
2	Arokiasamy	Cross-sectional	2018	6,262	14.8%	Low-Income	≥50 years	Grip strength
3	Avlund	Cohort	2011	573	14.5%	High-Income	70-year-olds, 75-year-olds (5 year follow up) and 80-year-olds (10 year follow up)	Mobility tiredness
4	Barros	Cohort	2013	1,635	25%	High-income	45-64 years	Chronic Obstructive Pulmonary Disease (COPD) related event

5	De Andrade	Cross-sectional	2013	1374	44.8%	Middle-income	≥60 years	Frailty
6	Gu	Cross-sectional	2019	3635	31.4%	Middle-income	≥65 years	Frailty
7	Heitmann	Cohort	2008	2,932	Not stated	High-income	35-65 years	Fatal and non-fatal cardiovascular disease (CVD), stroke and coronary heart disease (CHD)
8	Hewlett	Cross-sectional	2015	4,724	2.8%	Middle-Income	≥50 years	Subjective wellbeing
9	Huang and Cao	Cross-sectional	2022	16,209	20.3%	Middle-income	≥65 years	Functional disability
10	Kiesswetter	Cohort	2018	893	34.3%	High-income	55-80 years	Incident malnutrition
11	Koyama	Cross-sectional	2018	20,548	14.7%	High-income	≥65 years	Sleep duration

12	Koyanagi	Cross-sectional	2018	32,715	12.2%	Low- and middle-Income	≥50 years	Mild cognitive impairment
13	Koyanagi	Cross-sectional	2021	224,842	5.9%	Low- and Middle-Income	≥18 years	Subjective cognitive complaints (SCC)
14	Lee	Cross-sectional	2006	5,123	30.5%	High-income	≥60 years	Stroke history
15	Matsuyama	Cohort	2017	77,397	Males: 30.4% Females: 26.3%	High-income	≥65 years	Functional disability and all-cause mortality
16	Medina-Solis	Cross-sectional	2019	13,966	10.2%	Low-Income	35 to 98 years	Self-reported health status
17	Palmer	Cohort	2015	4,205	20.6%	High-income: six countries. Low-income: one country	≥18 years	All-cause cardiovascular mortality in people with end stage kidney disease treated with haemodialysis

18	Philips	Cohort	2021	7,343	17.1%	High-income	45-64 years	Undiagnosed diabetes mellitus
19	Ramsay	Cohort	2017	1,622	20%	High-income	71-92 years	Frailty
20	Ritchie	Cohort	2000	563	37%	High-income	≥70 years	Significant weight loss
21	Sanders	Cross-sectional	2016	7,305	20.6%	High-income	≥25 years	Signs and symptoms of obstructive sleep apnoea (OSA)
22	Smith	Cross-sectional	2022	237,023	Not stated (co-morbidity model)	Low- and Middle-Income	≥18 years	Sleep problems
23	Takata	Cross-sectional	2004	697	34.6%	High-Income	80 years old	Handgrip strength
24	Tyrovolas	Cross-sectional	2016	175,814	7.6%	Low-, middle- and high-Income	≥18 years	Depression and self- rated health

25	Vancampfort	Cross-sectional	2017	228,024	5.9%	Low- and Middle-Income	≥18 years	Physical activity
26	Vancampfort	Cross-sectional	2019	34,129	12.9%	Low- and Middle-Income	≥50 years	Weak handgrip strength
27	Vancampfort	Cross-sectional	2017	229,293	5%	Low- and Middle-Income	≥18 years	Perceived stress
28	Vancampfort	Cross-sectional	2017	181,845	6%	Low-, Middle- and High-Income	≥18 years	Anxiety
29	Vancampfort	Cross-sectional	2017	34,129	12.9%	Low- and Middle-Income	≥50 years	Highly sedentary behaviour
30	Vélazquez-Olmedo	Case- Cohort	2021	539	6.9%	Low-income	≥60 years	Frailty
31	Sabbah	Cross-sectional	2020	1,649	100%	High-income	≥18 years	All-cause mortality

32	Yu	Cross-sectional	2021	33,071	8.5%	High-income	≥20 years	All-cause and disease specific mortality
