# Supplementary material for: Impact of edentulism on community-dwelling adults in low-income, middle-income and high-income countries: a systematic review
Source: BMJ Open. 2024 Dec 4;14(12):e085479. doi: 10.1136/bmjopen-2024-085479 (PMC11624734; doi:10.1136/bmjopen-2024-085479)
Supplement: online supplemental file 9 [file bmjopen-14-12-s009.pdf]

Appendix 9: QualSyst Combined Scores Adapted from Kmet et al[17]

	First Author	Study Title	Q1	Q2	Q3	Q4	Q5	Q6	Q7	Q8	Q9	Q10	Q11	Q12	Q13	Q14	No (0)	Partial (1)	Yes (2)	N/A	total score	total score/(max score-N/A)
1	Vancampfort	The relationship between chronic physical conditions, multimorbidity and anxiety in the general population: A global perspective across 422 countries	2	2	2	1	N/A	N/A	N/A	0	2	2	2	1	2	2	1	1	9	3	18	18/22=0.82
2	Kiesswetter	Oral health determinants of incident malnutrition in community-dwelling older adults	2	1	1	1	N/A	N/A	N/A	0	2	2	2	1	2	2	1	4	6	3	16	16/22=0.73

3	Smith	Association between physical multimorbidity and sleep problems in 46 low- and middle-income countries	2	2	1	1	N/A	N/A	N/A	0	2	2	2	2	2	2	1	2	8	3	18	18/22=0.82
4	Vancampfort	Chronic physical conditions, multimorbidity and physical activity across 46 low and middle income countries	2	2	2	1	N/A	N/A	N/A	0	2	2	2	1	2	2	1	2	8	3	18	18/22=0.82
5	Koyanagi	Multimorbidity and Subjective Cognitive Complaints: Findings from 48 Low- and Middle-Income Countries of the World Health	2	2	2	1	N/A	N/A	N/A	0	2	2	2	2	2	2	1	2	8	3	19	19/22=0.86

		Survey 2002-2004																				
6	Velazquez-Olmedo	Oral health condition and development of frailty over a 12-month period in community-dwelling older adults	2	2	2	1	N/A	N/A	N/A	1	0	2	2	2	2	2	1	2	8	3	18	18/22=0.82
7	Vancampfort	Physical chronic conditions, multimorbidity and sedentary behavior amongst middle-aged and older adults in six low- and middle-income countries.	2	2	2	1	N/A	N/A	N/A	0	2	2	2	1	2	2	1	2	8	3	18	18/22=0.82
8	Koyama	Sleep duration and	2	2	1	1	N/A	N/A	N/A	1	2	2	2	1	2	2	0	4	7	3	18	18/22=0.82

		remaining teeth among older people																				
9	Heitmann	Remaining teeth, cardiovascular morbidity and death among adult Danes	2	2	1	0	N/A	N/A	N/A	2	2	2	2	2	2	2	1	1	9	3	19	19/22=0.86
10	Ramsay	Influence of Poor Oral Health on Physical Frailty: A Population-Based Cohort Study of Older British Men	2	2	1	1	N/A	N/A	N/A	1	2	2	2	1	2	2	0	4	7	3	18	18/22=0.82
11	Koyanagi	Chronic Physical Conditions, Multimorbidity, and Mild Cognitive Impairment in Low- and Middle-Income Countries	2	2	2	1	N/A	N/A	N/A	0	2	2	2	2	2	2	1	1	9	3	19	19/22=0.86

12	Vancampfort	Handgrip strength, chronic physical conditions and physical multimorbidity in middle-aged and older adults in six low- and middle income countries	2	2	2	1	N/A	N/A	N/A	1	2	2	2	1	2	2	0	3	8	3	19	19/22=0.86
13	Vancampfort	Perceived Stress and Its Relationship with Chronic Medical Conditions and Multimorbidity among 229,293 Community-Dwelling Adults in 44 Low- and Middle-Income Countries	2	2	2	1	N/A	N/A	N/A	1	2	2	2	1	2	2	0	3	8	3	19	19/22=0.86
14	Lee	The association	2	2	2	1	N/A	N/A	N/A	1	1	2	1	1	2	2	0	2	9	3	17	17/22=0.77

		between cumulative periodontal disease and stroke history in older adults																				
15	Barros	A Cohort Study of the Impact of Tooth Loss and Periodontal Disease on Respiratory Events among COPD Subjects: Modulatory Role of Systemic Biomarkers of Inflammation	2	2	2	0	N/A	N/A	N/A	2	2	2	2	2	2	2	1	0	10	3	20	20/22=0.91
16	Yu	Number of teeth is associated with all-cause and disease-specific mortality	2	2	2	1	N/A	N/A	N/A	2	2	2	2	2	2	2	0	3	8	3	21	21/22=0.95

17	Sabbah	Denture wearing and mortality risk in edentulous American adults: A propensity score analysis	2	2	2	2	N/A	N/A	N/A	2	2	2	2	2	2	2	0	0	11	3	22	22/22=1.0
18	Medina-Solis	Edentulism and other variables associated with self-reported health status in Mexican adults	2	2	2	1	N/A	N/A	N/A	0	2	2	2	2	2	2	1	1	9	3	19	19/22=0.86
19	Arokiasamy	Age, socioeconomic patterns and regional variations in grip strength among older adults (50+) in India: Evidence from WHO-SAGE	1	2	2	1	N/A	N/A	N/A	1	2	2	2	2	2	2	0	3	8	3	18	18/22=0.82
20	Matsuyama	Dental Status and Compression	2	2	2	1	N/A	N/A	N/A	1	1	2	2	2	2	2	0	3	8	3	19	19/22=0.86

		of Life Expectancy with Disability																				
21	Tyrovolas	Population prevalence of edentulism and its association with depression and self-rated health	2	2	2	1	N/A	N/A	N/A	0	2	2	2	1	2	2	1	2	8	3	18	18/22=0.82
22	Hewlett	Edentulism and quality of life among older Ghanaian adults	2	2	2	1	N/A	N/A	N/A	1	2	2	2	1	2	2	0	3	8	3	19	19/22=0.86
23	Sanders	Tooth loss and obstructive sleep apnea signs and symptoms in the US population	2	2	2	1	N/A	N/A	N/A	1	1	2	1	1	2	2	0	5	6	3	17	17/22=0.77
24	Palmer	Dental Health and Mortality in People With End-Stage Kidney	2	2	2	1	N/A	N/A	N/A	2	2	2	2	2	2	2	0	1	10	3	21	21/22=0.95

		Disease Treated With Hemodialysis : A Multinational Cohort Study																				
25	Philips	Periodontal disease, undiagnosed diabetes, and body mass index: Implications for diabetes screening by dentists	2	2	2	1	N/A	N/A	N/A	1	2	2	2	1	2	2	0	3	8	3	19	19/22=0.86
26	Avlund	Number of Teeth and Fatigue in Older Adults	2	2	2	2	N/A	N/A	N/A	1	0	2	0	2	2	2	2	1	9	3	17	17/22=0.77
27	Takata	Relationship of physical fitness to chewing in an 80-year-old population	2	2	2	1	N/A	N/A	N/A	1	0	2	2	2	2	2	1	2	8	3	18	18/22=0.82
28	Ritchie	Oral health problems and significant weight loss among	2	2	1	1	N/A	N/A	N/A	1	0	2	2	0	2	2	2	3	6	3	15	1/252=0.68

		community-dwelling older adults																				
29	DeAndrade	Relationship between oral health and frailty in community-dwelling elderly individuals in Brazil	2	2	2	0	N/A	N/A	N/A	1	1	2	2	2	2	2	1	2	8	3	18	18/22=0.82
30	Gu	Association between the number of teeth and frailty among Chinese older adults: a nationwide cross-sectional study. BMJ Open 9.	2	2	2	1	N/A	N/A	N/A	1	2	2	1	2	2	2	0	3	8	3	19	19/22=0.86
31	Albani	Associations of poor oral health with frailty and physical functioning in the oldest old: results from two	2	2	2	1	N/A	N/A	N/A	1	1	2	2	2	2	2	0	3	8	3	19	19/22=0.86

		studies in England and Japan. BMC Geriatr.																				
32	Haung	Tooth loss trajectories and their association with functional disability among older chinese adults: results from the chinese longitudinal healthy longevity survey	2	2	2	1	N/A	N/A	N/A	1	2	2	2	2	2	2	0	2	9	3	20	20/22=0.91
