# Supplementary material for: Impact of edentulism on community-dwelling adults in low-income, middle-income and high-income countries: a systematic review
Source: BMJ Open. 2024 Dec 4;14(12):e085479. doi: 10.1136/bmjopen-2024-085479 (PMC11624734; doi:10.1136/bmjopen-2024-085479)
Supplement: online supplemental file 10 [file bmjopen-14-12-s010.pdf]

Appendix 10: Data Extracted from Included Studies

Broad Outcome Area	First Author	Specific Outcome	Measurement Tool	Metric Used to Report Results	Reported Results	Relative Effect	Adjustments	QualSyst Score
**Frailty**	**Matsuyama**	Functional Disability	New acceptance for Long- Term Care Insurance Level 2 or more and determined by assessment of physical and cognitive impairments using the Certification Committee of Needed Long-Term Care	Hazard Ratio	Compared to edentulous patients, participants with 1-9, 10-19 and ≥20 teeth had statistically significant lower risks of transitioning from healthy status to disabled	**Age adjusted only: ≥20 teeth Men:** HR = 0.42 (0.36-0.49) **Women**: HR = 0.52 (0.44-0.61) P<0.05<br><br>**10-19 teeth Men:** HR 0.58 (0.50-0.67) **Women**: 0.70 (0.60-0.81)<br><br>**1-9 teeth Men:** 0.74 (0.65-0.84) **Women**: 0.72 (0.63-0.82)	In the fully adjusted model, age, denture use, socioeconomic status, health behaviour and health status were adjusted for	86%

						**Fully adjusted: ≥20 teeth Men: HR =** 0.52 (0.44–0.61) **Women**: HR= 0.58 (0.49–0.68) P<0.05<br><br>**10-19 teeth Men:** 0.65 (0.56-0.76) **Women**: 0.75 (0.86)<br><br>**1-9 teeth Men:** 0.77 (0.67-0.88) **Women**: 0.73 (0.64-0.83)		
	**Vancampfort**	Low Physical Activity	Self-reported answers to elements of The International Physical Activity Questionnaire	Odds Ratio	Edentulism was overall significantly associated with low physical activity in the overall sample. When stratified by age, the only age groups that remained statistically	Fully adjusted model:<br><br>Edentulism:<br><br>**All age groups:** OR = 1.46 (1.30- 1.64) P<0.0001	Adjustments were made for age, sex, education, wealth and country. Sleep problems mediated 5.9% for	82%

					significant were the 35-49 age group and the ≥65 years	**Age 18-34:** OR= 1.24 (0.92-1.68) P=0.1568<br><br>**Age 35-49 years**: OR= 1.37 (1.06-1.76) p= 0.0159<br><br>**Age 50-64 years:** OR= 1.20 (0.98-1.46)P= 0.0758<br><br>**Age ≥65 years:** OR= 1.22 (1.03-1.45) P= 0.0197	edentulism, mobility mediated 8.7%, depression mediated 6.7% and pain mediated 9.2%, all of which were statistically significant P<0.05	
	**Vancampfort**	Weak Handgrip Strength	Smedley's hand dynamometer	Odds Ratio	There was a association between edentulism and weak handgrip strength	**Fully adjusted model:** Edentulism OR = 1.23 (1.03-1.47) P<0.05	Adjusted for by country, sex, age, wealth, marital status, BMI, physical activity,	86%

							depression and smoking status	
	**Ramsay**	Frailty after 3 years follow up	Fried Frailty Phenotype: self-reported and physical examination	Odds Ratio	Compared to individuals with ≥1 teeth, edentulous men have increased likelihood of frailty in both age and fully adjusted models	Compared to individuals with ≥1 teeth, edentulous participants:<br><br>**Age adjusted only:**<br>OR = 1.84 (1.37-2.48). P<0.05<br><br>**Fully adjusted model:**<br>OR =1.63 (1.18-2.23) P<0.05	Full adjustment for by social class, age, smoking, diabetes history, cardiovascular disease history and use of medications that cause xerostomia.	82%

	**Velazquez-Olmedo**	Frailty after 12 months	Fried Frailty Phenotype: self-reported and physical examination	Odds Ratio	Edentulous participants had an increased risk of the development of frailty over 12 months compared to those with acceptable oral health	Compared to those with acceptable oral health**,**<br><br>Edentulous participants:<br><br>**Unadjusted model:**<br>OR = 4.1 (1.9-8.4) P<0.001<br><br>**Fully adjusted model:**<br>OR 2.3 (1.0-5.1) P = 0.05	Fully adjusted for age, sex, education level, paid employment, marital status, comorbidities, depressive symptoms, use of oral health services, cognitive impairment and nutritional status	82%

	**Arokiasamy**	Handgrip Strength	Smedley's hand dynamometer	Probability	**Men**: There was negatively associated with grip strength in edentulous individuals compared to dentate individuals.<br><br>**Women**: There was a prediction of reduced grip strength in edentulous participants compared to dentate individuals.<br><br>This relationship was not was not statistically significant in the female model	Fully adjusted:<br>**Men**:<br>β coefficient = -1.73 (-2.48- -0.99) P<0.001<br><br>**Women**:<br>β coefficient = -0.43 (-1.00- 0.13) P= 0.136	Adjusted for by marital status, height and place of residence	82%
	**Huang**	Functional Disability	Performances of Activities of Daily Living (ADL) and Instrumental ADL (IADL)	Odds Ratio	There was a statistically significant association between edentulism and functional disability compared to those with mild tooth loss	**Adjusted for follow-up time:**<br>**ADL Disability:** OR 3.15 (2.58, 3.86)<br><br>**IADL Disability:** OR	In the fully adjusted model, residence, marital status, smoking status, drinking status,	91%

						3.57 (3.00, 4.24)<br><br>**Fully adjusted model:**<br>**ADL Disability:** OR 3.25 (2.58, 4.09)<br><br>**IADL Disability:** OR 3.60(2.93-4.42)	denture use, and multimorbidity	
	**De Andrade**	Frailty	Frailty was defined on the basis of five characteristics: weight loss, weakness, slowness, exhaustion, and low level of physical activity. Participants with three or more characteristics were classified as frail, with one or two	Odds ratio	Participants with 21 or more teeth had a had a 75% lower chance of being frail than those who were edentulous. Results were significant only in Comparison between edentulous and individuals with 21 or more teeth. There was no statistical significance in comparison	Compared to people with 0 teeth, odds of frailty:<br><br>**Individuals with 1-10 teeth:**<br>OR 0.86 (0.41–1.82) P=0.69<br>Individuals with 11-20 teeth:<br>OR 0.32 (0.09-1.17) P=0.08	Socioeconomic and general factors known to be related to the outcome.	82%

			as prefrail, and with zero as non-frail.<br>and low level of physical activity)		between edentulous individuals and individuals with 1-10 teeth and those with 11-20 teeth	**Individuals with 21 or more teeth**: OR 0.25 (0.07–0.91) P=0.04		
	**Avlund**	Fatigue	Mobility Tiredness (Mob-T) Scale on six mobility activities: walking outdoors in good weather, climbing stairs, walking outdoors in poor weather, going outdoors, walking indoors and transferring	Odds ratio	Compared to individuals with 20 or more teeth, tooth loss was associated with the onset of fatigue in old age but this association was attenuated more than 10% when socioeconomic factors and smoking were controlled for. Evidence was noted in in the crude model, however significance was lost when adjustments were made	**Unadjusted model:**<br><br>**Age 70: OR:1.96(1.24 -3.10) P<0.05**<br><br>**Age 75: OR:2.47(1.45 -4.23) P<0.05**<br><br>**Age 80: OR: 2.21(1.18-4.14) P<0.05**<br><br>**Fully adjusted model:**<br><br>**Age 70: OR**	Education, smoking, income and comorbidity.	77%

						**1.23 (0.74-2.12) P<0.05**<br><br>**Age 75: OR 1.88(0.97-3.64) P>0.05**<br><br>**Age 80: OR 1.24 (0.57-2.67) P>0.05**		

	**Takata**	Physical fitness	Handgrip strength, leg extensor strength, leg extensor power, stepping rate, one-leg standing time	Odds ratio	Compared to edentulous people, people with 1-9 teeth had increased odds of higher handgrip strength, leg extensor strength, isokinetic extension power and stepping rate. No relationships were significant. Compared to edentulous people, people with 20 or more teeth had reduced handgrip strength, stepping rate and isokinetic leg extension but higher leg extension power. No results were statistically significant.Classification based on tooth number, did not show significant relationship with any physical fitness	Compared to people with 0 teeth, **People with 1-9 teeth:**<br><br>Handgrip strength: OR 1.236 (0.656–2.329)<br><br>Leg extensor strength: OR 1.100 (0.596–2.030)<br><br>Isokinetic leg extension power: OR 1.219 (0.645–2.303)<br><br>Stepping rate: OR 1.131 (0.686–1.863<br><br>One- legged standing time:	Height, body weight, gender, SBP, serum albumin concentration, FSG, back pain, smoking, alcohol drinking, marital status, regular medical treatment and regular exercise	82%

					test<br>by logistic<br>regression analysis	OR 1.341<br>(0.841–<br>2.140)<br><br>**People with 10-19 teeth:**<br>Handgrip strength: OR 1.158 (0.564–2.377)<br><br>Leg extensor strength:<br>OR 1.365<br>(0.682–<br>2.732)<br><br>Isokinetic leg extension power:<br>OR 0.964<br>(0.475–<br>1.958)<br><br>Stepping rate:<br>OR 1.299<br>(0.747–2.258<br><br>One- legged standing time:<br>OR 1.191		

						(0.717–1.978)<br><br>**People with 20 or more teeth:**<br>Handgrip strength: OR 0.795 (0.355–1.779)<br><br>Leg extensor strength:<br>OR 1.817 (0.842–3.919)<br><br>Isokinetic leg extension power:<br>OR 0.859 (0.387–1.905)<br><br>Stepping rate:<br>OR 0.833 (0.451–1.538)<br><br>One- legged standing		

						time: OR 1.380 (0.774–2.459)<br><br>No relationships were significant<br><br>P>0.05		
	**Ritchie**	Significant weight loss	With gender, income, advanced age, and baseline weight controlled for, edentulousness remained an independent risk factor for significant weight loss (odds ratio 1.63 for 4% weight loss and 2.03 for 10% weight loss)	Odds ratio	With gender, income, advanced age, and baseline weight controlled for, edentulousness remained an independent risk factor for significant weight loss (odds ratio 1.63 for 4% weight loss and 2.03 for 10% weight loss)	**Unadjusted model:**<br><br>Edentulousness prediction of 4% weight loss over 1 year:<br>OR 1.78 (1.24, 2.56).<br><br>Edentulousness prediction of 10% weight loss over 1 year:<br>OR: 2.08 (1.16, 3.70)<br><br>**Fully adjusted**	Gender, income, advanced age, and baseline weight adjusted for. Addition of other potential confounders (depression, smoking, alcohol use, physical activity) did not change the estimates.	68%

						**multiple logistic regression model**: Edentulousness prediction of 10% weight loss over 1 year: OR: 2.03 (1.05, 3.96) P<0.05		
	**Gu**	Frailty (and number of teeth)	Frailty Index	Odds ratio	After adjusting for the covariates, older adults with 0 teeth had significantly higher odds of frailty than those with >20 teeth. The same associations were noted in adults with 1-10 and 11-20 teeth, however significance was lost in the 11-20 teeth group when fully adjusted.	Compared to individuals with >20 teeth<br><br>**Unadjusted model:**<br><br>**0 teeth:** OR 4.76 (3.66 to 6.20) P<0.001<br><br>**1-10 teeth:** 3.42 (2.62-4.46) P<0.001<br><br>**11-20 teeth**:	Demographic characteristics including age, sex, co-residence, marital status, years of education and financial support), body mass index (BMI) and health behavior's (i.e, smoking, drinking and exercise	91%

						1.82 (1.34-2.47) P<0.001<br><br>**Fully adjusted model:**<br><br>**0 teeth**: OR: 2.07 (1.53-2.80) P<0.001<br><br>**1-10 teeth:** 1.77 (1.31-2.38) P<0.001<br><br>**11-20 teeth**: 1.30 (0.93-2.08) P=0.122		

	**Albani**	Frailty	The Fried Frailty Phenotype	Odds ratio		**Adjusted for age only:**<br><br>**Newcastle 85+ Study:**<br><br>**F**or edentulous patients compared to 1 or more teeth<br>**Frailty:** OR 1.53 (1.11-2.12)<br>**Mobility Limitations:** OR: 1.48 (1.12-1.97)<br>**Weak grip strength:** OR: 1.37 (1.01-1.85)<br>**Slow gait speed:** OR: 1.60 (1.19,2.16)<br><br>**For TOOTH study:**<br>**Frailty:** OR:	Adjusted for age, sex, BMI, alcohol intake, smoking status, social class, cardiovascular disease, diabetes, hypertension, neuropsychiatric disease and other health conditions (cerebrovascular disease, respiratory disease, musculoskeletal disease, and cancer diagnosis in the last 5 years).	86%

						1.63 (1.06,2.55)<br>**Mobility Limitations:** OR 1.49 (0.87, 2.54)<br>**Weak grip strength:** OR 1.07 (0.71-1.60)<br>**Slow gait speed:** OR 1.32 (0.86-2.02)<br><br>**Fully adjusted model:**<br><br>**Newcastle 85+ Study:** For edentulous patients compared to those with one or more teeth.<br>**Frailty:** OR: 1.41 (0.95-2.07)<br>**Mobility**		

						**limitations:** OR 1.32 (0.95-1.83)<br>**Weak grip strength** OR: 1.42 (1.00-2.02)<br>**Slow gait speed:** OR: 1.50 (1.05-2.14)<br><br>**For TOOTH study:**<br>**Frailty:** OR: 1.67 (1.05-2.67)<br>**Mobility Limitations:** OR 1.54 (0.85- 2.78)<br>**Weak grip strength:** OR 1.02 (0.66-1.57)<br>**Slow gait speed:** OR 1.35 (0.86-2.12)<br><br>**P>0.05/<0.05**		

**General Health**	**Barros**	COPD Related Events	COPD hospitalisation with ICD9 code entered in discharge notes or included as primary, secondary or underlying cause of death	Hazard Ratio	Compared to individuals with COPD and periodontal health, edentulous patients who had COPD had an increased risk of experiencing a COPD related event	**Unadjusted model:** HR = 4.66 (3.21-6.78) P<0.05<br><br>**Fully adjusted model:** HR = 2.28 (1.46-3.56) P<0.05	Adjusted for by smoking, age, BMI, diabetes history, education, income and research centre/ race	91%
	**Philips**	Undiagnosed Diabetes Mellitus	Self-reported no diagnosis of diabetes	Odds Ratio	Edentulous participants were significantly associated with undiagnosed diabetes mellitus (UDM) in the Periodontal Profile Classes system model compared to Stage 1 healthy participants	**Fully adjusted model:**<br><br>**Edentulousness:** OR = 1.87 (1.27-2.75) P<0.05	Adjusted for by sex, age, race, BMI, lipid profile and Atherosclerosis Risk in Communities (ARIC) centre%	86%

	**Lee**	Stroke History	Self-reported history of stroke	Odds Ratio	Completely edentulous participants and patients with one edentulous arch had a significant association with having a stroke history compared to individuals with teeth in both arches. This study noted that it was unclear if periodontal disease, a precursor to edentulism was a risk factor for stroke or a disease marker.	Compared to individuals with teeth present in both arches<br><br>**Fully adjusted model:**<br><br>**Completely edentulous**: OR = 1.49 (1.07-2.08)<br><br>**Edentulous in one arch:** OR 1.49(1.07-2.08)<br><br>P=0.029	Adjusted for by age and smoking. Unclear if periodontal disease is a risk factor for stroke or if could be a marker of the disease	77%

	**Heitmann**	Fatal and non-fatal Cardiovascular disease (CVD), stroke and coronary heart disease (CHD)	Information on fatal and non-fatal CVD, CHD and stroke obtained from the Patient Registry of Hospital Discharges, Cause of Death Register and Central Person Register	Hazard Ratio	Compared to dentate individuals, edentulous participants had a three-time increased risk of stroke and a 50% increased risk of cardiovascular disease. Risk for coronary heart disease was not significant after adjustments	**First quintile* compared to the fifth quintile (27-32 teeth for men, 26-32 teeth for women), edentulous participants had:**<br><br>**Model 1:**<br>**Stroke:** HR = 3.71 (1.88-7.31)<br>**Cardiovascular disease:** HR = 1.98 (1.41-2.78)<br>**Coronary heart disease:** HR = 2.68 (1.60-4.47)<br>P<0.05<br><br>**Fully adjusted**	**Model 1:** Adjusted for age, gender and whether tooth counting was performed 1987/88 or 1993/94:<br><br>**Model 5:** Adjusted for age, gender and whether tooth counting was performed 1987/88 or 1993/94, education, smoking, age, diabetes, alcohol. BMI, systolic blood pressure and education	86%

						**Model 5:**<br>**Stroke: HR = 3**.25 (1.48-7.14)<br>**Cardiovascular disease:** HR = 1.50 (1.02-2.19)<br>**Coronary heart disease:** HR = 1.31 (0.74-2.31)<br>P<0.05<br><br>***First quintile had 0 teeth for all analyses except for associations with CVD for men where the first quintile was 0-4 teeth		

	**Sanders**	Signs and symptoms of obstructive sleep apnoea (OSA)	Self-reported questions adapted from the Sleep Habits Questionnaire and Blood Pressure Questionnaire. Considered high risk of OSA if ≥2 positive responses to questions	Prevalence ratios	Compared to fully dentate individuals, edentulous participants (and those experiencing tooth loss) had a two times higher risk for OSA symptoms. This remained statistically significant after adjustment.	Compared to people with a full dentition:<br><br>**Unadjusted model:**<br><br>**Edentulousness:**<br>PR 2.16 (1.62-2.88) P<0.001<br><br>**9-31 lost teeth:**<br>PR 1.81 (1.46-2.23) P<0.001<br><br>**5-8 lost teeth:**<br>PR 1.44 (1.26-1.65) P<0.001<br><br>**Fully adjusted model:**<br><br>**Edentulousn**	Adjusted for by BMI, age, sex and ethnicity	77%

						**ess:**<br>PR 1.61<br>(1.11-2.33)<br>P=0.014<br><br>**9-31 lost teeth:**<br>PR 1.36<br>(1.06-1.73)<br>P=0.016<br><br>**5-8 lost teeth:**<br>PR 1.25<br>(1.07-1.46)<br>P=0.006		

**Mental Health**	**Koyanagi**	Mild Cognitive Impairment	Assessed by using recommendations by National Institute on Aging and Alzheimer's Association.	Odds Ratio	Edentulous patients had increased odds of mild cognitive impairment:	**Fully adjusted model:**<br><br>**Overall sample:**<br>OR = 1.24 (1.03- 1.48) P<0.05<br><br>**Age 50-64:**<br>OR 1.23 (0.95-1.58)<br><br>**Age ≥65 :**<br>OR 1.21 (0.95-1.55)	Adjusted for by age, sex, education, level, BMI, alcohol use, smoking, wealth, physical illness and depression	86%
	**Koyanagi**	Subjective Cognitive Complaints	Self-reported Subjective Cognitive Complaint within the last 30 days	Mean SCC scores	Edentulism was significantly associated with higher mean SCC scores than dentate individuals	**Fully adjusted model:**<br>**β coefficient** = 2.43 (1.19, 3.67)<br>P<0.05	Adjusted for by age, education, wealth, sex, other chronic conditions and country.	86%

	**Vancampfort**	Perceived Stress (score ranged from 0 to 100 with higher scores indicating higher levels of perceived stress)	Self-reported answers to the Perceived Stress Scale	The association between edentulism and perceived stress was estimated by multivariable linear regression of the mean perceived stress scores. β coefficient for perceived stress	Edentulous participants had higher mean perceived stress scores compared to dentate individuals in the multivariable linear regression model. This was not statistically significant. When poverty (lowest individual level wealth quintile) was added to the model, the association became statistically significant.	Associations between chronic medical conditions and perceived stress (outcome)<br><br>**Edentulism: Fully adjusted model:**<br>β coefficient = 0.65 (-0.54-1.83)<br>P= 0.2846<br><br>Effect of interaction between individual level poverty and edentulism:<br><br>**Main effect: β coefficient** = -0.17(-1.53-1.19)<br>P<0.0001.	Adjusted for by country, age, sex, education, individual level wealth and other illnesses	86%

						**+ Poverty: β coefficien**t = 2.05(1.17-2.94) P<0.0001.<br><br>**Interaction: β coefficien**t = 3.36(0.78-5.94) P=0.0109.<br><br>*****poverty was defined as the lowest countryside individual wealth quintile		
	**Vancampf ort**	Anxiety	Self-reported answers to questions on anxiety symptoms within the last 30 days.	Odds Ratio	There was initially an association between edentulism and increased odds of anxiety. This became insignificant when depression was added to the multivariable	**Model 1:** OR = 1.14 (1.01, 1.29) P<0.05<br><br>**Model 2:** OR = 1.13 (0.97, 1.31) P<0.05	**Model 1: A**djustment for age, sex, wealth and country:<br><br>**Model 2:** Fully adjusted for age, sex, wealth,	82%

					logistic regression model		country and depression	
	**Tyrovolas**	Depression (and Self-Rated Health)	Self-reported lifetime experience of depression and/ or Depression within the last 12 months based on symptom duration and persistence using the Diagnostic and Statistical Manual of Mental Disorders Fourth edition (DSM-IV Algorithm	Odds Ratio	**Age<50 years:** There was a statistically significant association between edentulism and depression.<br><br>**Age ≥50 years:** There was no significant association between edentulism and depression<br><br>**This study considered that younger people may be more likely to experience depression with edentulism due to the stigma of tooth loss at a younger age and potential**	Fully adjusted: **Age<50 years:** OR = 1.57 (1.23-2.00) P=0.003<br><br>**Age ≥50 years**: OR = 1.03 (0.84-1.26) P= 0.789	Edentulism was only statistically associated with depression in the under 50 age group. Adjustments made for chronic conditions, country, smoking, wealth, disability, alcohol consumption, age and education.	82%

					**impact on self-esteem**			

**Mortalit y**	**Sabbah**	All-cause mortality	Status of death obtained from National Death Index database	Hazard Ratio	**Weighted samples: All-cause mortality:** Edentulous denture wearers had significantly reduced risk of all-cause mortality compared to edentulous non-denture wearers.<br><br>**Cardiovascular mortality:** Edentulous denture wearers had reduced risk of cardiovascular death compared to edentulous non-denture wearers but it was non-significant.<br><br>**Cancer mortality:** Edentulous denture wearers had reduced risk of cancer mortality compared to edentulous non-denture wearers	**Full adjusted model:**<br><br>**Weighted samples:** Compared to non- denture wearers, denture wearers had:<br><br>**All-cause mortality:** HR = 0.85 (0.73-0.98) P<0.05<br><br>**Cardiovascular mortality:** HR = 0.84 (0.67-1.06) P>0.05<br><br>**Cancer mortality:** HR = 0.87 (0.64-1.17). P>0.05<br><br>**Matched samples:**	Twenty-seven covariates adjusted for	100%

					but it was non-significant.<br><br>The difference in mortality rate between non-denture wearers and denture wearers was 11.1(3.6-18.6)	**C**ompared to non- denture wearers, denture wearers had:<br><br>**All-cause mortality:** HR = 0.79 (0.68-0.92) P<0.05<br><br>**Cardiovascular mortality:** HR = 0.81 (0.66-1.00) P>0.05<br><br>**Cancer**		

						**mortality:** HR = 0.78 (0.58-1.04). P>0.05<br><br>**A**ll-cause mortality rate for the weighted sample for non-denture wearers 76.6(68.1-86.1) was higher than denture wearers 65.5(61.9-69.3). Similar was noted in the matched sample with an all-cause mortality rate of 74.4(60.2-91.2) in non-denture wearers compared to 65.7(31.9-69.7) in		

						denture wearers.		
	**Matsuyama**	All-cause mortality,	Mortality data from Long-term Care Insurance Database	Hazard Ratio	Compared to edentulous patients, participants with ≥20 teeth had lower risks of transitioning from healthy status to dead.	**Age adjusted only**<br>Compared to people with 0 teeth, the risk of transitioning from healthy to dead were as follows:<br><br>**≥20 teeth**<br>**Men:** HR 0.48 (0.41-0.55)<br>**Women:**<br>HR 0.62 (0.51-0.76)<br><br>**10-19 teeth**<br>**Men:** HR 0.63 (0.55-0.73)<br>**Women:** HR 0.76 (0.63-0.92)<br><br>**1-9 teeth**<br>**Men:**<br>0.76 (0.67-0.87)	Age, denture use, socioeconomic status, health behaviour and health status were adjusted for.	86%

						**Women:** 0.76 (0.64-0.91)<br><br>**Fully adjusted:** Compared to people with 0 teeth, the risk of transitioning from healthy to dead were as follows:<br><br>**≥20 teeth**<br>**Men:** HR =0.58 (0.50-0.68)<br>**Women** HR = 0.70 (0.57-0.85)<br><br>**10-19 teeth**<br>**Men:** 0.71 (0.62-0.82)<br>**Women:** 0.81 (0.67- 0.98)<br><br>**1-9 teeth**		

						**Men:** 0.80 (0.70-0.91) **Women:** 0.77 (0.64-0.92)<br><br>Among individuals aged ≥85 years, 75-79 years and 65-69 years dentate people had a longer life expectancy, healthy life expectancy and shorter life expectancy with disability for men and women compared to edentulous patients. For example, life expectancy for people with ≥ 20		

						teeth was 57 days for men and 15 days longer for women, healthy life expectancy was 92 days longer for men and 70 days for women) and shorter life expectancy with disability (92 days shorter for men, 70 days for women) than edentulous participants.		

	**Yu**	All-cause mortality	NHANES definition for mortality cases	Hazard Ratio	Compared to dentate individuals, edentulous participants had statistically significant increased risks of all-cause mortality. However, greater risks were seen in participants with 0-9 teeth across all models of adjustment. However, data for edentulous disease-specific mortality was grouped with dentate data (0-9 teeth).	**Compared to dentate participants, edentulous patients had all- cause mortality hazard ratios of:**<br><br>**Model 1:**<br>HR = 1.53 (1.42-1.64) P>0.001<br><br>**Model 2:**<br>HR = 1.24 (1.15-1.34) P>0.001<br><br>**Model 3:**<br>HR = 1.35 (1.17-1.57) P>0.001	**Model 1:** Adjustment for age, sex, race, smoking adjustment.<br><br>**Model 2:** Adjustment for age, sex, race, smoking, education, income-poverty ratio, BMI, physical activities, hypertension, CVD, diabetes, stroke.<br><br>**Model 3:** Adjustment for age, sex, race, smoking, education, income-poverty ratio, BMI, physical activities, hypertension, CVD,	95%

							diabetes, stroke and femoral neck bone mineral density	

	**Palmer**	All-cause and cardiovascular mortality in people with end stage kidney disease treated with haemodialysis	All-cause and disease specific mortality was extracted from a patient status monitor database	Hazard Ratio	Among adults with end- stage kidney disease. **All-cause:** Edentulousness was associated with increased risks of mortality compared to dentate individuals **Cardiovascular death:** Edentulousness was associated with increased risk of death compared to dentate individuals. For both outcomes, edentulism had reduced risk of death compared to individuals with greater than or equal to 14 decayed, missing and filled teeth.	Edentulism associated with: **All-cause mortality** HR 1.29 (1.10-1.51) P<0.05 **Cardiovascular death:** HR 1.28 (1.02-1.60) P<0.05 Adjusted associations of edentulism with all-cause mortality) and cardiovascular mortality were stronger in adults <60 years (all-cause mortality HR 1.74(1.15-2.62) and	Adjustments made for age, race, smoking history, sex, medical history, dialysis vintage, blood pressure, serum phosphorus, haemoglobin, mean arterial blood pressure	95%

						cardiovascular mortality HR 2.48(1.47-4.17)) compared to older adults (all-cause mortality HR 1.33(1.15-1.55) and cardiovascular mortality 1.29(1.05-1.60))		

**Quality of Life**	**Hewlett**	Subjective wellbeing	Self-reported. Measured with the use of elements of the World Health Organization Quality of Life (WHOQOL) instruments and single item measures, transformed into a 0-100 scale. Lower scores indicate a better quality of life.	Mean WHOQOL score	Edentulous participants had lower levels of subjective wellbeing compared to dentate individuals in terms of energy levels, health, self and life satisfaction, relationships, living conditions, ability to perform tasks and quality of life.<br><br>Edentulism was observed to be associated with significantly lower levels of SWB among older adults using both the single-item and multiple-item measures (WHOQOL). The WHOQOL correlated positively and strongly with the single-item	Mean WHOQOL score:<br><br>**Dentate Patients**: 54.09<br><br>**Edentulous patients**: 61.60<br><br>P=0.00<br><br>After adjustment for age, sex, education, chronic disease condition and income in the multivariate analysis, the difference between edentulous and dentate participants became statistically insignificant.	Age, sex, income, education and the diagnosis of a chronic disease	86%

					measure<br><br>Being edentulous was significantly associated with a higher mean WHOQOL score thus a higher level of dissatisfaction with one's life. The association between edentulous patients reporting moderate self-rated health compared to dentate patients was not significant.<br><br>There was a statistically significant increased likelihood of edentulous patients reporting bad or very bad self-rated health.	Happiness showed no association with edentulism		

	**Medina-Solis**	Self-reported health status	Self-reported health. Participants were asked to grade their health as very good, good, moderate, bad and very bad health	Odds Ratio	The association between edentulous patients reporting moderate self-rated health compared to dentate patients was not significant.<br><br>There was a statistically significant increased likelihood of edentulous patients reporting bad or very bad self-rated health.<br><br>Age modified the association between being edentulous and poor/very poor self-reported health. Interaction 1.02 (95%CI 1.01-1.03). Poor and very poor self-reported health among edentulous	Bivariate logistic regression:<br><br>**Edentulous patients likelihood reporting moderate vs good self-rated health compared to non-edentulous patients:** OR 1.73 (1.39-1.81) P<0.001<br><br>**Edentulous patients likelihood of reporting bad or very bad vs good self-rated health compared to non-edentulous patients:** OR 3.26	Adjusted for by age, sex, occupation, education, socio-economic level, disability, physical activity, chronic disease, alcohol use and BMI.	86%

					patients were higher in younger participants.	(2.51-4.25). P<0.001<br><br>Fully adjusted model:<br><br>**Edentulous patients likelihood reporting moderate vs good self-rated health compared to non-edentulous patients**:<br>OR 2.05 (0.69-6.08) P>0.05<br><br>**Edentulous patients likelihood of reporting bad or very bad vs good self-rated health compared to non-edentulous**		

						**patients:**<br><br>OR 17.56 (4.22-73.03). P<0.001		
	**Tyrovolas**	Self-rated health	Self-reported asking participants ‘in general, how would you rate your health today?’	Odds Ratio	Edentulism was significantly associated with poor self-rated health in the under 50 age group. In the over 50 age group, the association was not significant	Fully adjusted model:<br><br>**Age<50 years:** OR 1.38 (1.03-1.83). P=0.028<br><br>**Age ≥50 years:** OR 1.04 (0.89-1.23). P=0.605	Adjusted for age, sex, education, alcohol use, wealth, smoking, country and chronic conditions.	82%

**Nutrition**	**Kiesswetter**	Incident Malnutrition	Self-reported BMI and involuntary weight loss	Hazard Ratio (HR)	Compared to individuals with greater than 7 teeth in both the mandible and maxilla, edentulous individuals had a statistically insignificant increased risk of 9-year incident malnutrition for both crude and adjusted models. Study indicates that xerostomia and poor oral health in combination with edentulism contributes to the development of malnutrition.	Compared to individuals with greater than 7 teeth in both the mandible and maxilla<br><br>**Edentulous participants:**<br><br>**Unadjusted model:**<br>HR 1.33 (0.87-2.04) P=0.186<br><br>**Adjusted for age, gender, education and income:**<br>HR 1.16 (0.73-1.84) P= 0.540<br><br>Participants with 1-7 teeth in at least one jaw:<br><br>**Unadjusted model:**	Adjusted for age, gender, education, income	73%

						HR 1.15<br>(0.73-1.81)<br>P=0.544<br><br>**Adjusted for age, gender, education and income:** HR 1.35<br>(0.83-2.17)<br>P= 0.225		
**Health Behaviour**	**Vancampfort**	Sedentary Behaviour	Self-reported answers to questions on sedentary behaviour	Odds Ratio	There was an association between edentulism and highly sedentary behaviour across all age groups but it was not significant	**Fully adjusted model:**<br><br>**Overall sample:** OR 1.11 (0.86-1.43)<br>P>0.05<br><br>**50-64 years:** OR 0.87<br>(0.61-1.26)<br>P>0.05<br><br>**≥65 years:** OR 1.14<br>(0.84-1.54)<br>P>0.05	Adjusted for by age, wealth, education level, sex, setting, living situation and employment status	82%

	**Smith**	Sleep problems	Self-reported difficulties falling asleep, waking up frequently, waking up too early	Odds Ratio	There was a statistically significant association between edentulism and sleep problems	**Fully adjusted model:**<br><br>**Edentulism:** OR = 1.19 (1.04-1.36) P<0.05<br><br>**24% of association explained by pain, 21% explained by anxiety, 11.2% explained by depression and 10.4% explained by stress**	Adjustments made for age, education, sex, smoking, alcohol, wealth, physical activity, country and obesity.	82%

	**Koyama**	Sleep duration	Self-reported asking individuals how many hours they slept in a day	Relative Risk Ratio	Edentulous participants had significantly higher relative risk ratio for shorter sleep duration (≤ 4 hours), longer sleep duration ≥10 hours) and 8 hours sleep compared to people with ≥20 teeth,	**Fully adjusted model:**<br><br>**Compared to people with ≥20 teeth, edentulous people had:**<br><br>**For shorter sleep (≤4 hours): RRR =** 1.43 (1.07-1.90) P<0.05<br><br>**5 hours:** RRR= 1.04 (0.87-1.24) P>0.05<br><br>**6 hours:** RRR= 0.97 (0.85-1.11) P>0.05<br><br>**8 hours:** RRR= 1.28 (1.13-1.46) P<0.05	Adjusted for age, sex, BMI, annual household income, educational attainment, smoking history, activities of daily living, frequency of going out and Geriatric Depression Scale	82%

						**9 hours:**<br>RRR= 1.58 (1.29-1.93) P<0.05<br><br>**For long sleep(≥10 hours):** RRR = 1.75 (1.40-2.19) P<0.05		
