# Supplementary material for: Impact of edentulism on community-dwelling adults in low-income, middle-income and high-income countries: a systematic review
Source: BMJ Open. 2024 Dec 4;14(12):e085479. doi: 10.1136/bmjopen-2024-085479 (PMC11624734; doi:10.1136/bmjopen-2024-085479)
Supplement: Supplementary file 11 [file bmjopen-2024-085479.reviewer_comments.pdf]

PEER REVIEW HISTORY

BMJ Open publishes all reviews undertaken for accepted manuscripts. Reviewers are asked to complete a checklist review form and are provided with free text boxes to elaborate on their assessment. These free text comments are reproduced below.

ARTICLE DETAILS

Title (Provisional)

The impact of edentulism for community-dwelling adults in low-, middle- and high-income countries: a systematic review

Authors

Hunter, Emma; De Moura Brito, Luciana; Piyasena, Prabhath; Petrauskiene, Egle; Congdon, Nathan; Tsakos, Georgios; Virgili, Gianni; Mathur, Manu; Woodside, Jayne; Leles, Cláudio; McKenna, Gerald

---

VERSION 1 - REVIEW

**Reviewer**	**1**
**Name**	**Polido, Waldemar**
**Affiliation**	**Indiana University, Oral and Maxillofacial Surgery**
**Date**	**20-Mar-2024**
**COI**	**No competing interests.**

Thank you for doing this review and addressing this important issue of edentulism. With the growth of restorative techniques including implants, less and less effort has been made to preserve teeth and improve global oral health, even in developed countries. This review highlights the importance of teeth in quality of life, productivity and overall health. It can serve as a record for those in the oral health leadership positions in different parts of the world to invest and optimize oral health care, preservation of teeth and rehabilitation of those that can not maintain their teeth.

**Reviewer**	**2**
**Name**	**McGrath, Colman P. J.**
**Affiliation**	**Univ Hong Kong, Dentistry**
**Date**	**26-Mar-2024**
**COI**	**None**

Abstract: Clear

Introduction: A sound rationale for the review is provided in terms of need to review evidence on global impacts of edentulism. It may be useful to include in the introduction findings form other reviews on impact of edentulism – global burden etc.

Methods: The review benefits from being a prior registered review. The search strategy and section is clearly described. Risk of bias clearly outlines and appropriately used. Synthesis without meta-analyses outlined (SiIM).

Results: A clear narrative synthesis is provided in terms of frailty, general health, mental health, mortality, quality of life, nutrition and health behaviours.

Discussion:

A reasonable discussion is provided in terms of key findings. Limitations of the review could be expanded upon.

VERSION 1 - AUTHOR RESPONSE

Requested change by editorial team	Action taken
1. PBP missing:<br>- Please provide a point-by-point response to the Editor's comments and reviewer's comments.	Addressed- table included to provide response to the editor's comments and a table to respond to the peer review comments has been included in the response.
2. Guarantor in Contributorship statement missing:<br>- BMJ's Contributorship statement policy indicates that each statement must make clear who is responsible for the overall content as guarantor (https://authors.bmj.com/policies/bmj-policy-on-authorship/#contributorship%20statement). Please ensure the guarantor has been named in the contributorship statement accordingly. Examples include:<br>'Author name / Author initials are the guarantor'<br>'Author name / Author initials acted as guarantor'<br>'Author name / Author initials are responsible for the overall content [as guarantor].'	Addressed. EH is the guarantor to the article
3. Marked copy:<br>- Aside from the clean copy, please also provide a marked copy of your manuscript with 'tracked changes' and upload it under the file designation 'Main document-marked copy'. This is to show all the changes you have made for your paper.	A marked and clean copy have been uploaded
4. Required figure/s format:	Figures have been added as both Word and PDF documents

- Figures can be supplied in TIFF, JPG or PDF format (figures in document, excel or powerpoint format will not be accepted), we also request that they have a resolution of at least 300 dpi and 90mm x 90mm of width.	
5. Required Supplementary format:<br>- Please re-upload your Supplementary files in PDF format.	All COI forms, prisma checklist and tables and figures have been reuploaded as pdf
6. Table citation order:<br>- You have cited Table 3 rights after Table 4 which makes your citations incorrect. Please review again your main document and ensure that all references will be cited and will appear in numerical order.	Amended- table 3 should have been cited earlier. This has now been rectified.
7. Author's name mismatched:<br>- The author “Virgilli, Gianni” in your main document is registered as “Virgili, Giann” in ScholarOne. Please ensure that the author has same registered name.	Author name should be: Gianni Virgili. This is now reflective of the Scholar One account.
8. Reference citation order:<br>- You have cited reference #2 rights after reference #3 which makes your citations incorrect. Please review again your main document and ensure that all references will be cited and will appear in numerical order.	Addressed. Citations reordered in introduction and in results and discussion section of manuscript.
9. Contributorship statement mismatch:<br>- Please ensure that your “Contributorship statement” in your main document and ScholarOne submission system is the same.	This has been amended.
10. Incomplete contributorship statement:<br>- Please provide a more detailed contributorship statement. It needs to mention all the names/initials of authors along with their specific contribution/participation for the article. *Manu Raj Mathur and Cláudio Leles missing in contributorship statement	This has been amended. The contributions of Cláudio Leles and Manu Raj Mathur have been added to the manuscript.

Peer review comment	Response
Reviewer: 1<br>Dr. Waldemar Polido, Indiana University<br>Comments to the Author:<br>Thank you for doing this review and addressing	Thank you for the positive comments on the maniscript and the team are grateful for the acknowledgement of the potential impact of the review for policy makers.

this important issue of edentulism. With the growth of restorative techniques including implants, less and less effort has been made to preserve teeth and improve global oral health, even in developed countries. This review highlights the importance of teeth in quality of life, productivity and overall health. It can serve as a record for those in the oral health leadership positions in different parts of the world to invest and optimize oral health care, preservation of teeth and rehabilitation of those that can not maintain their teeth.	
Reviewer: 2<br>Dr. Colman P. J. McGrath, Univ Hong Kong<br>Comments to the Author:<br>Abstract: Clear<br>Introduction: A sound rationale for the review is provided in terms of need to review evidence on global impacts of edentulism. It may be useful to include in the introduction findings form other reviews on impact of edentulism – global burden etc.<br>Methods: The review benefits from being a prior registered review. The search strategy and section is clearly described. Risk of bias clearly outlines and appropriately used. Synthesis without meta-analyses outlined (SiIM).<br>Results: A clear narrative synthesis is provided in terms of frailty, general health, mental health, mortality, quality of life, nutrition and health behaviours.<br>Discussion:<br>A reasonable discussion is provided in terms of key findings. Limitations of the review could be expanded upon.	Thank you for your comments on the review. In order to address the comment to strengthen the limitations of the study, we have reflected on our manuscript and developed our limitations further in line 510, 532 and 537 on our marked copy.<br><br>We appreciate the suggestion to add findings from other reviews on the impact of edentulism. In our introduction, we have added an additional explanation to this section of the review in line 289.

VERSION 2 - AUTHOR RESPONSE

Revision	Change made
*The main manuscript file has been uploaded as a PDF - we required this to be supplied in Word format, in order to be able to utilise the file to generate proofs. Please resupply the main manuscript in Word format.	Clean and marked copy of manuscript (with in-text tables) uploaded as word documents. A copy of the manuscript without in text tables has also been provided.
*The figure should be removed from the main manuscript file - this should only be uploaded as a separate 'Figure' file and not included in the main	The PRISMA flow chart has been uploaded in a separate pdf as a figure file.

manuscript. The figure title/caption should be included at the very end of the main manuscript (after the reference list) and not included in the 'Figure' file.	The PRISMA flow chart has been removed from the main manuscript file.<br><br>A figure list has been added to the end of the manuscript, after the reference list. Figure 1: PRISMA flow chart has been added to this list.
*Thank you for including a copy of the PRISMA checklist with your submission (Appendix 1). As the page numbers will not be accurate in the final published version, you may wish to revise this checklist to indicate sections rather than pages. Alternatively, you can remove any citations of the checklist (appendix 1) from the manuscript and upload the existing file as a research checklist [for the editors and reviewers only] rather than as supplemental material (so that it is not part of the final publication; note that if you do this any other supplementary files/appendices may need to be renamed, with citations in the text updated accordingly). If you maintain the PRISMA checklist as an appendix cited in the main manuscript, please upload the checklist as a 'Supplemental material' file rather than a 'Research checklist' file and ensure the file name includes 'Appendix 1' for clarity.	The PRISMA checklist has been revised to remove reference to page numbers and instead, sections have been included.<br><br>The PRISMA checklist has been uploaded as a research checklist and removed from the appendices list. The citations for appendices have been updated in the main text.<br><br>All other appendices have been re-uploaded in the correct citation order
*Please combine all the search strategy appendix files (appendix 2) into a single PDF file.	All search strategies have been combined into one PDF file and uploaded
*Please revise the fourth bullet point in the 'Strengths and limitations of this study' section so that it consists of a single sentence.	The fourth bullet point in the strength and weaknesses section has been rephrased into one sentence

1) Please ensure that author affiliations (including departments) are identical in both the Title Page and submission system. Any secondary affiliations must also be listed in both.	This has been addressed.
2) Please include figure legends at the end of your main document file.	Following the previous request, I have included this below the reference list as requested. I would be grateful if you could please specify if there is an issue with how this has been done.
3)) Tables should be in Word format and placed in the main text where the table is first cited. Please remove your table files, embedding them into the main text where the table is first cited. Tables should be created within the word document, not inserted as an image.	I have included my tables in the file named: Main document- marked copy with in text tables and figures 130924.docx. I had previously been asked to upload all figures separately in a PDF and I have uploaded the tables in a PDF for completeness.

4) Your tables are too large to be included in the manuscript, specifically tables 1, 2, and 3. Please either reduce their size (maximum 2 pages / 8 columns) or upload them as supplemental material.	Table 1,2,3 changed to supplementary files appendix 8,9,10. References to this changed in the manuscript and in the prisma checklist.
5) Please ensure that all authors are included in your competing interest statement. For further information please see https://authors.bmj.com/policies/bmj-policy-on-authorship/	I had written 'All other authors declare no competing interests.' I have now changed to All other authors (GMK, JW, NC, CL, GV, EP, LM, MM) declare no competing interests.
